# Review of the Nordic *Gymnocheta* Robineau-Desvoidy (Diptera, Tachinidae) with report of two species new to Europe

**DOI:** 10.3897/zookeys.1053.52761

**Published:** 2021-08-02

**Authors:** Jaakko Pohjoismäki, Christer Bergström

**Affiliations:** 1 University of Eastern Finland, Department of Environmental and Biological Sciences, P.O. Box 111, FI-80101 Joensuu, Finland University of Eastern Finland Joensuu Finland; 2 Säves väg 10, SE-75263 Uppsala, Sweden Unaffiliated Uppsala Sweden

**Keywords:** DNA barcoding, Co1, Ernestiini, Fennoscandia, *
Gymnocheta
*, new species, species diversity, synonymy, Tachininae, type specimens

## Abstract

The genus *Gymnocheta* Robineau-Desvoidy, 1830 (Diptera, Tachinidae) has until now been represented by two species in Europe, *G.viridis* (Fallén, 1810) and *G.magna* Zimin, 1958. Two species are newly recorded from Finland and Sweden, *Gymnochetalucida* Zimin, 1958 and *G.zhelochovtsevi* Zimin, 1958, both previously known only from the Russian Far East and Japan. These four European species are redescribed and illustrated, including the first description of the female of *G.zhelochovtsevi*. A key is provided to seven of the eight described species of Palaearctic *Gymnocheta*. The holotype of *G.viridis* was examined and found to differ from the present concept of the species, instead matching the concept of the more recently described *G.magna*. In the interests of nomenclatural stability, the two names are maintained in their current usage pending a request to the International Commission on Zoological Nomenclature to replace the current holotype of *G.viridis* with a neotype that corresponds to the long-established concept of that species.

## Introduction

**Figure 1.**
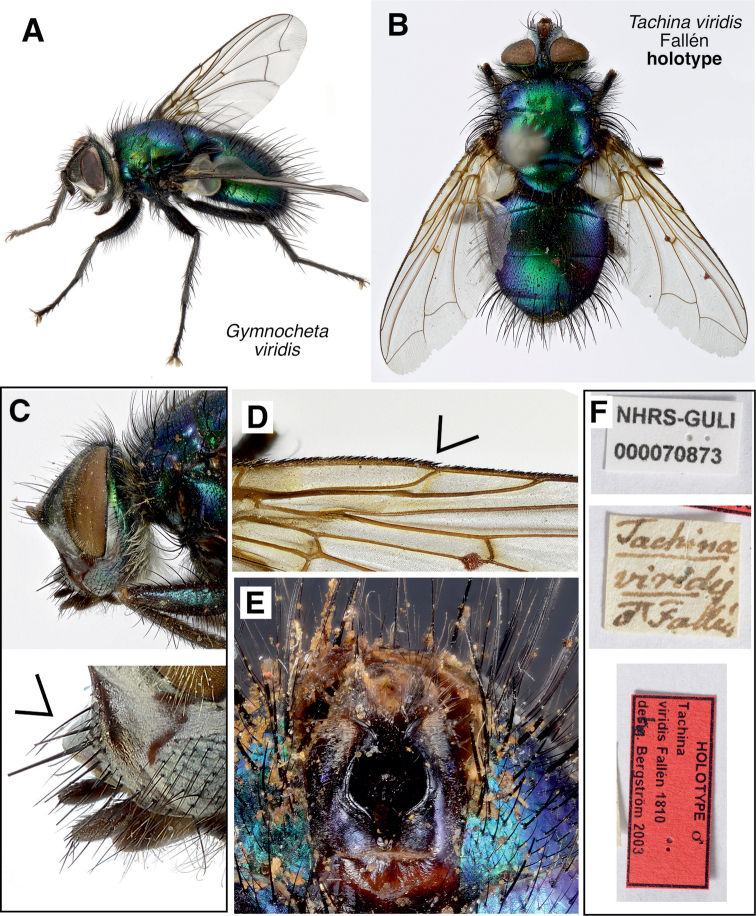
*Gymnocheta* spp. habitus **A** habitus *Gymnochetaviridis* ♂ **B–E***Tachinaviridis* Fallén, holotype specimen **B** dorsal view **C** head in lateral view. Note the protruding lower facial margin (arrowhead) **D** detail of the right wing edge. Note the reduced costal spine (arrowhead) **E** sternite 5 of the holotype **F** labels associated with the holotype. All photographs by Göran Liljeberg.

The genus *Gymnocheta* Robineau-Desvoidy, 1830 constitutes a morphologically homogenous group within the large and multiform tribe Ernestiini and includes medium to large (5.0–13.0 mm), semi-robust tachinids, easily recognisable by their metallic green colouration (Fig. 1), which can convert to hues of metallic blue, purple and red in living as well as in dead specimens. The only other genus of Tachininae in Europe with metallic green members is *Chrysosomopsis* Townsend (Mesnil 1971; Zeegers et al. 2016), which is easily recognised from *Gymnocheta* by their yellow (vs. black) palps and three (vs. four) pairs of postsutural dorsocentral setae. For further diagnostic characters of *Gymnocheta* see Mesnil (1972), Tschorsnig and Richter (1998), and Cerretti et al. (2012).

*Gymnocheta* is currently known from eight species in the Palaearctic Region (Herting and Dely-Draskovits 1993) and four species in the Nearctic Region (O’Hara and Wood 2004). Interestingly, *G.viridis* (Fallén) was the only Palaearctic species recognised before the work of Zimin (1958). He increased the number of species to seven of which three (*G.flamma* Zimin, *G.mesnili* Zimin and *G.porphyrophora* Zimin) were restricted to China and two (*G.lucida* Zimin and *G.zhelochovtsevi* Zimin) to the Russian Far East. The only widespread species described in the publication was *G.magna* Zimin, with a distribution from Central Europe to Japan (Richter 2004). Only one Palaearctic *Gymnocheta* species has been discovered after Zimin’s paper, *G.goniata* Chao, 1979 from China.

During a DNA barcoding study of Finnish Tachinidae (Pohjoismäki et al. 2016), we discovered that the Finnish specimens identified as *Gymnochetaviridis* (Fallén) clustered under two different barcode index numbers (BINs, Ratnasingham and Hebert 2013), BOLD:ACF3891 and BOLD:ACA6555. While some of the specimens had an identical mitochondrial cytochrome oxidase subunit 1 (Co1) gene sequence with the Central European *G.viridis*, some others had a sequence that was distinct from it as well as from that of *Gymnochetamagna* Zimin, the only other known European species of the genus. A closer examination revealed that the differing specimens were also morphologically dissimilar to *G.viridis* and had been collected exclusively from bog habitats, suggesting that the BIN split could be due to a genuine species difference. In fact, once the species difference was realised, additional specimens of this unknown species were recognised from Sweden and originated from similar habitats as in Finland. While investigating specimens in Finnish collections, we also noted the existence of a second unreported and not barcoded species in southern Finland, which differed from the other three species by its wide frons in the male and characteristic shape of the male syncercus.

A survey of the potential species candidates for the two unknown Nordic species among the described Palaearctic species found tentative matches with the descriptions of *G.lucida* and *G.zhelochovtsevi*. The species identities were later confirmed by comparing Nordic specimens with Russian and Japanese material. As an aid to future researchers, redescriptions of both species (originally described in Russian), including also the previously unknown female of *G.zhelochovtsevi*, are provided and full redescriptions of the other European species are given. Also included are notes on the known biology of the species and a key for the identification of the Palaearctic *Gymnocheta* species (not including the little-known *G.goniata*).

An examination of the holotype of *Tachinaviridis* Fallén, 1810 (Fig. 1B–F) revealed that it is the same species as *Gymnochetamagna* Zimin, 1958. Normally this synonymy would require the concept of *Gymnochetaviridis* to change to that of *G.magna* and the species currently called *G.magna* would be given a replacement name, in accordance with the provisions of the International Code of Zoological Nomenclature (1999). However, compliance with the Code would cause a great deal of confusion over the identity of the well-established name *G.viridis*. In the interests of nomenclatural stability, we follow Article 75.6 of the Code (“Conservation of prevailing usage by a neotype”) in maintaining prevailing usage of the names *G.viridis* and *G.magna* and will request the International Commission on Zoological Nomenclature to set aside under its plenary power the holotype of *Tachinaviridis* Fallén and designate in its place a neotype that corresponds to the current interpretation of the species.

## Materials and methods

**Figure 2.**
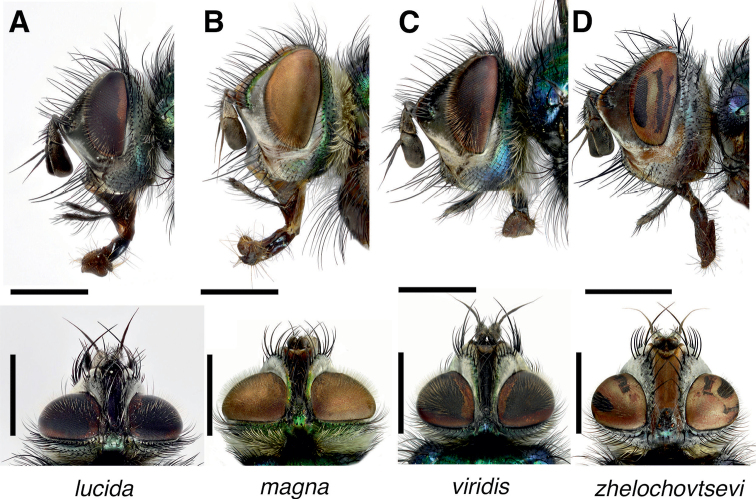
*Gymnocheta* spp. male heads in lateral (upper panels) and dorsal (lower panels) view **A***Gymnochetalucida* Zimin **B***G.magna* Zimin **C***G.viridis* (Fallén) **D***G.zhelochovtsevi* Zimin. Note the wide frons and strong outer vertical bristles. Scale bars: 1 mm. All photographs by Göran Liljeberg.

**Figure 3.**
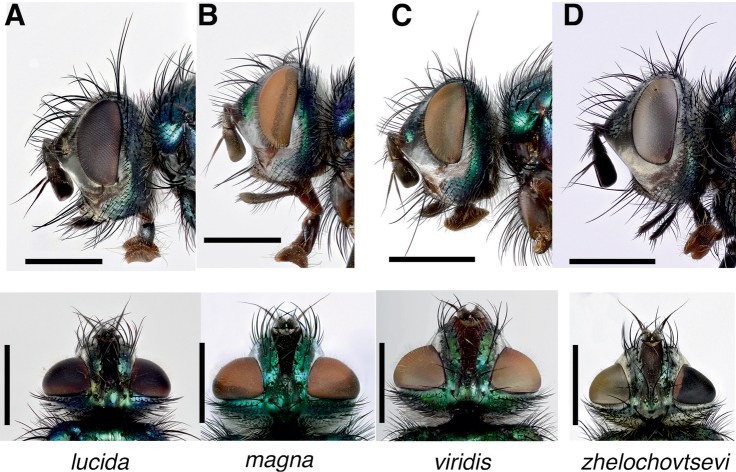
*Gymnocheta* spp. female heads in lateral (upper panels) and dorsal (lower panels) view **A***Gymnochetalucida* Zimin **B***G.magna* Zimin **C***G.viridis* (Fallén) **D***G.zhelochovtsevi* Zimin. Scale bars: 1 mm. All photographs by Göran Liljeberg.

Male and female terminalia were dissected and prepared for examination following the method described by O’Hara (2002). Terminalia are preserved in glycerol in a small plastic vial pinned together with the specimen. External morphological images were taken with a Nikon D2X (Figs 1A, 2B–D) or a Nikon D800 (Figs 1B, 2A, 3A–D) digital camera mounted to a bellow and a macro-optical tube. Helicon Focus, a program that combines the focused areas from the several partially focused images, was used to create one completely focused image. The images were cropped and colour- and contrast-enhanced, but not otherwise manipulated. The material examined is deposited in the following collections (acronyms are used in the text):

**AHC** Private collection of A. Haarto, Mietoinen, Finland;

**BLKU**Biosystematics Laboratory, Kyushu University, Fukuoka, Japan;

**CBC** Private collection of C. Bergström, Uppsala, Sweden;

**JPC** Private collection of J. Pohjoismäki, Joensuu, Finland;

**MZH**Finnish Museum of Natural History, Zoological Museum, University of Helsinki, Helsinki, Finland;

**NHRS**Swedish Museum of Natural History, Stockholm, Sweden;

**REC** Private collection of Roger Engelmark, Gubböle, Sweden;

**TMNH** Tampere Museum of Natural History, Tampere, Finland;

**ZIN**Zoological Institute, Russian Academy of Sciences, St. Petersburg, Russia;

**ZMLU**Museum of Zoology, Lund University, Lund, Sweden.

Label data are given verbatim using the following symbols: / for the end of a line and beginning of the next; // at the end of a label and beginning of the next (from top to bottom on the same pin).

The classification follows Herting and Dely-Draskovits (1993), apart from using *Chrysosomopsis* Townsend instead of *Chrysocosmius* Bezzi (see Mesnil 1971; Zeegers et al. 2016) and *Panzeria* instead of *Ernestia*, *Appendicia*, and *Eurithia* (Cerretti et al. 2012; O’Hara and Henderson 2020). The morphological terminology used in this study follows Cummin and Wood (2017) and Tschorsnig (1985) for some features of the male terminalia.

Cytochrome oxidase subunit 1 (**Co1**) DNA barcoding was performed as a part of the Tachinidae project of Finnish Barcode of Life initiative (**FinBoL**). The 5´-terminal part of Co1 was amplified using the routine barcoding primers LepF1 and LepR1 (Hebert et al. 2004). The results of the study as well as release of the barcode sequences have been described in detail in Pohjoismäki et al. (2016). All sequences used in this study are available in the GenBank (https://www.ncbi.nlm.nih.gov/genbank/) and the access IDs listed with the listing of each specimen (see above). Other members of the Ernestiini (sensu Herting 1984), *Panzeriapuparum* (Fabricius) (GenBank ID KX844044), *P.rudis* (Fallén) (GenBank ID KX844400), *P.truncata* (Zetterstedt) (GenBank ID KX843735), *P.anthophila* (Robineau-Desvoidy) (GenBank ID KX844254), *P.caesia* (Fallén) (GenBank ID KX843746), *P.connivens* (Zetterstedt) (GenBank ID KX843746), *P.vivida* (Zetterstedt) (GenBank ID KX843724), *Cleonicecallida* (Meigen) (GenBank ID KX843803), *C.keteli* Ziegler (GenBank ID KX844536), *C.nitidiuscula* (Zetterstedt) (BOLD sample ID JP2016_1), and *Zophomyiatemula* (Scopoli) (GenBank ID KX844031), were included for comparison.

Sequence comparisons were performed using MUSCLE alignment (Edgar 2004) and Bayesian inference phylogenetic tree generated using MrBayes 3.2. (Ronquist et al. 2012), applying GTR substitution model with gamma-distributed rate variation across sites and a proportion of invariable sites, and 1,000,000 MCMC generations. The tree was visualised using FigTree 1.4.4. (Rambaut 2009).

### Material examined

For clarity, the information for the material on *Gymnochetalucida*, *G.magna*, *G.viridis*, and *G.zhelochovtsevi* is given under the corresponding species descriptions. In addition to these species, the following non-European species is also included to confirm the identity of the Nordic *G.zhelochovtsevi*.

***Gymnochetaporphyrophora* Zimin, 1958**

**Figure 4.**
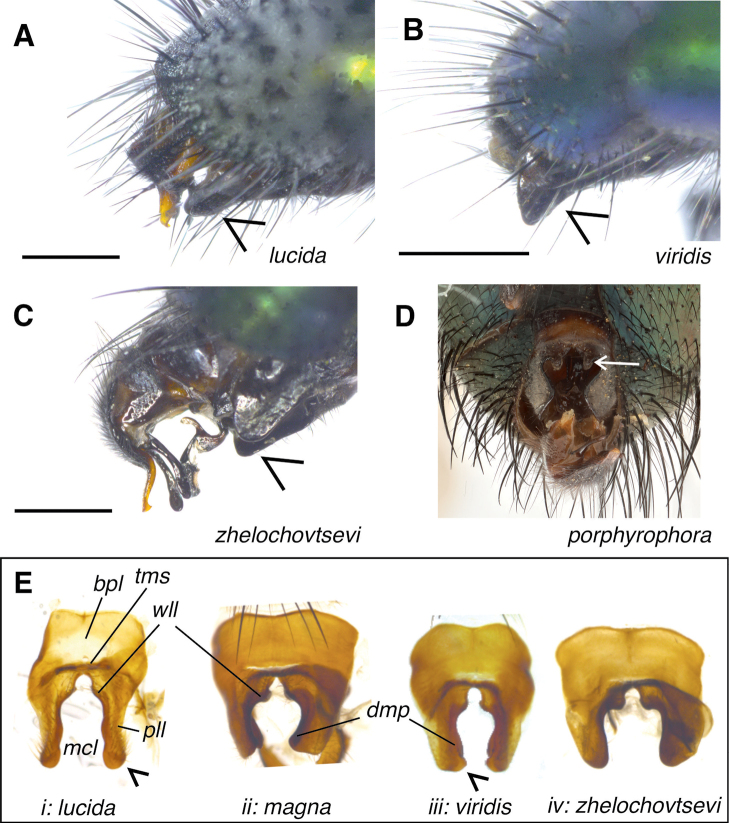
Fifth sternites of *Gymnocheta* spp. males **A***Gymnochetalucida* male abdomen. Note the narrow profile of the posterior edge of the fifth sternite (arrow) **B** the edge of the fifth sternite in *G.viridis* is bulky and protruding beyond the tip of the abdomen, clearly recognisable from the side **C** the posterolateral lobe of *G.zhelochovtsevi* is somewhat subrectangular as in *G.magna*, but more rounded in profile. Note the apical hook of the syncercus **D** in contrast to *G.zhelochovtsevi*, posterior edge of fifth sternite in *G.porphyrophora* with well-developed median lobes (arrow) **E** dissected fifth sternites of Nordic *Gymnocheta* spp. in comparison: i) *G.lucida*, ii) *G.magna*, iii) *G.viridis* and iv) *G.zhelochovtsevi*. A distinct dorsomedial process present in *G.magna* and *G.viridis* Abbreviations: **bpl**–basal plate; **dmp**–dorsomedial process; **mcl**–median cleft; **pll**–posterolateral lobe; **tms**–transversal median stripe; **wll**–wart-like lobe (or median lobe). Photograph **D** by V. Neimorovets, rest by J. Pohjoismäki.

**China**: (1♂, 1♀) 1♂: Сев. предгорье......., Грум-Гржимайло, 14.V.1890 [Northern Foothills....... (not readable), Grum-Grzhimailo, 14.V.1890]; 1♀: р. Сэрг-чу, 13800, близ Желтей, Тибет, Козлов, конец V.1901 [river Sarg-chu, 13800, near Yeltei, Tibet, Kozlov, end V.1901]. Coll. A. Semenov-Tian-Shansky. [ZIN]. Examined from high quality photographs, including the terminalia (Fig. 4D). **Nepal**: (1♂, 1♀) 1♂: (E. NEPAL) / Thurupka (2600 m), 27° 36´ N, 87° 36´ E - - - / Topke Gola (3700 m) / 27° 38´ N, 87° 35´ E // June 12, 1972 / H. Shima leg. / Kyushu Univ. Col. // *Gymnochaetopsis* / *porphyrophora* Z. (handwritten) / det. H. Shima 2020 [BLKU]; 1♀: (E. NEPAL) / Thudam (3500 m) / 27° 45´ N, 87° 32´ E / July 8, 1972 / Malaise trap (4) / Kyushu Univ. Col. *Gymnochaetopsis* / *porphyrophora* Z. (handwritten) / det. H. Shima 2020 [BLKU].

## Taxonomy

### Genus Gymnocheta

Taxon classificationAnimaliaDipteraTachinidae

Robineau-Desvoidy, 1830

56915026-6BE6-59C1-82A1-F51417567B47

Gymnocheta Robineau-Desvoidy, 1830: 371 (also subsequently spelled Gymnochaeta, unjustified emendation). Type species: Tachinaviridis Fallén, 1810 (as viridis Meigen), by monotypy (see also O’Hara et al. 2009: 142).

#### Redescription

**Figure 5.**
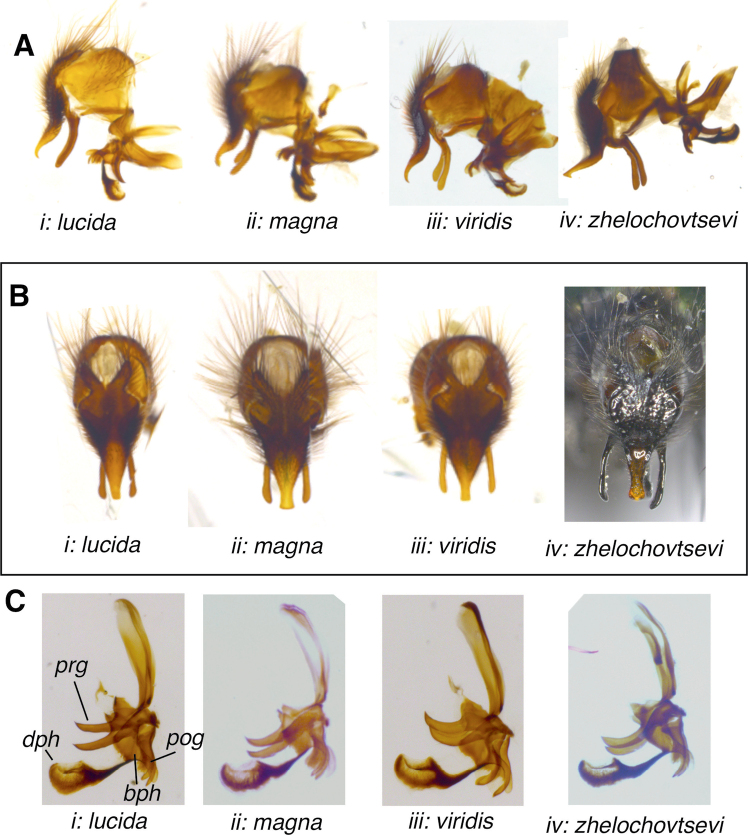
Comparison of *Gymnocheta* spp. male terminalia **A** lateral view of the terminalia: **i***G.lucida***ii***G.magna***iii***G.viridis* and **iv***G.zhelochovtsevi* – note the apical hook of the syncercus **B** dorsal view of the terminalia: **i***G.lucida***ii***G.magna***iii***G.viridis* and **iv***G.zhelochovtsevi*, same specimen as in Fig. 4C**C** aedeagus and gonites of **i***G.lucida***ii***G.magna***iii***G.viridis* and **iv***G.zhelochovtsevi*. Abbreviations: **bph**–basiphallus; **dph**–distiphallus; **prg**–pregonite; **pog**–postgonite. All photographs by J. Pohjoismäki.

**(European species). Male** (Figs 1, 2, 4, 5).

***Colouration* (Fig. 1)**: The colour described here is based on freshly collected specimens. In older specimens the black or dark brown colour is often changed to brown, red brown, or beige brown. Head to a varying extent covered with microtomentum, which affects how clearly the metallic green ground colour shines through, the latter characteristic of the genal dilation, fronto-orbital plate, and occiput but sometimes hard to recognise, as the interpretation depends on the direction of the incidence of light: the specimens should be viewed from different angles. Facial plate light brown to black with or without a metallic green spot at lower margin. Parafacial black but in older specimens narrowly reddish-brown along the ptilinal fissure. Genal groove reddish brown. Frontal vitta dark brown to black. Occiput and postgena with white to greyish white hairs. Antenna matt dark brown or black due to the greyish microtomentum, pedicel sometimes lighter apically, arista black. Palpus black or dark brown in older bleached specimens. Clypeus (sometimes with a metallic green tint) and prementum black, labella from dark brown to beige brown.

Thorax and abdomen with different shades of metallic green but often also partly blue, purple or red. In our European species *G.magna* and *G.viridis* show a bright metallic ground colour, *G.lucida* an olive green and *G.zhelochovtsevi* a dark green colour, the shine depending on the direction of the light. Scutum, when viewed from the side and slightly from behind, with four black longitudinal stripes of microtomentum, changing from grey to purple depending on the direction of the incident light; presutural area with the medial stripes narrow and widely separated, the lateral stripes are wedge-shaped and reach the level of posthumeral seta. In aged specimens, caught late in the season, the microtomentum can be worn off and give the specimens a polished appearance on scutum. Proepisternum black and densely covered with microtomentum or with the metallic green colour slightly subshiny. Legs normally extensively black or dark brown, which can change to lighter brown in aged specimens; fore coxa in anterodorsal region and sometimes also femora with remnants of metallic green or blue tint, covered with light grey microtomentum. Wing veins black and/or brownish black, wing membrane with a brownish tinge. Wing membrane around crossvein r-m sometimes narrowly infuscate. Tegula and basicosta dark brown to black. Halteres brown but stalk and knob partly blackish. Calypters greyish white, edge for the most part beige but inner edge distinctly infuscate, with a white fringe.

***Head* (Figs 1, 2)**: Head in profile angularly protruding at level of antennal insertion, width of parafacial at this level ca. 0.6–0.8 × the horizontal eye diameter. Frons in dorsal view at narrowest point, 0.3–0.7 × the width of an eye. Frontal vitta gradually tapering toward ocellar tubercle in *G.magna* and *G.viridis*, tapering toward middle (sometimes narrowest here) and then parallel-sided in *G.lucida* or wide and parallel-sided in *G.zhelochovtsevi*. Fronto-orbital plate with a row of 10–14 medioclinate strong frontal setae and some additional setulae, uppermost setula tiny and sometimes slightly reclinate; 3–5 setae extend on upper part of parafacial, reaching the middle of the pedicel with the row curving laterally, and here sometimes accompanied by some setulae; frontal plate outside the frontal row of setae with sparsely short and tiny setulae. Height of face 0.8–0.9 × the length of frons. Gena in profile at narrowest point, 0.3–0.4 × as high as vertical eye diameter. Lower anterior area of genal groove in front of genal dilation bare or rarely with some minute setulae. Vibrissa normally well developed and slightly shorter than height of face, inserted at the level of the lower facial margin. In some specimens of *G.lucida* there is no distinct vibrissa but two or three equally strong supravibrissal setae. Facial plate slightly convex between the antennae and except for *G.magna* hardly visible in profile; lower facial margin protruding particularly in the middle but except for *G.magna* only vaguely visible in profile. Chaetotaxy variable: Facial ridge with 1–4 strong and 0–3 additional thinner supravibrissal setae on lowest quarter (length of them at least equal to narrowest width of parafacial) and 2–5 thin and short setulae. Ridge below vibrissa with 3–7 strong subvibrissal setae continuous with the genal setae, longest 0.5–0.6 × the length of vibrissa. Ocellar tubercle with the ocelli forming a slightly pointed or in some specimens an almost equilateral triangle, two strong lateroproclinate ocellar setae inserted between anterior ocellus and posterior ocelli, accompanied by a tuft of thin setulae (sometimes one or two pairs of additional shorter setae may be present). Inner vertical setae crossed, at least subequal in length to the ocellar setae. Outer vertical setae less developed and subequal with the adjacent postocular setae, except for *G.zhelochovtsevi* where they are almost subequal with the inner vertical. Postocular setae long and thin, apically pronouncedly bending forward over the eyes except for *G.zhelochovtsevi*. Occiput evenly convex, normally with a pair of postocellar setae, rarely missing; in upper part with 2(3) somewhat irregular rows of short black setae behind the postocular row of setae. Palpus slightly clavate at tip, subequal to the length of the antennae, with black setulae. Antenna: Scape erect. Pedicel subtriangular and with one elongate seta. First flagellomere relatively short, in profile usually subrectangular or slightly widened towards apex, but sometimes thick and extended or flat and truncate; maximal width in profile 0.8–1.3 × as wide as parafacial at narrowest point and 1.4–1.8 × as long as pedicel. Arista at first sight bare but at higher magnification pubescence, widened in its proximal 1/4 to almost 2/3, and gradually tapering to apex. Eyes densely covered with long hairs, white with a yellowish tint, hairs slightly shorter in *G.zhelochovtsevi*.

***Thorax* (Fig. 1)**: Proepisternum bare. Prosternum bare or with tiny setulae present. Postpronotal lobe with 4(5) strong setae, the three basal arranged in an almost right angle, a slightly weaker anterior seta anterior or anterolateral to the inner basal seta. Chaetotaxy of scutum variable: 3(2)+3 acrostichal, 3(2)+4 dorsocentral and 1+3 intra-alar setae. Posthumeral seta inside and in front of the presutural intra-alar seta, both close to edge of postpronotum; 1+4 supra-alar setae, presutural supra-alar inserted in the middle between transverse suture and postpronotum; first postsutural supra-alar seta at least subequal with the notopleural setae, shorter than first postsutural intra-alar seta; two notopleural setae, two strong postalar setae. Ground vestiture on scutum (consisting of thin setulae) dense and erect, longest setulae subequal to the shortest setae. Katepisternum with three setae (2+1), rarely four, present. Anepimeron with 2(3) strong setae subequal to the strongest katepisternal setae. Katepimeron bare or with up to five tiny setulae in anterior 1/2. Scutellum also with a rather variable chaetotaxy; normally with four or five pairs of strong marginal setae, sometimes mixed with some shorter and weaker marginal setae/setulae, almost horizontal with the plane of scutellum, apical setae missing, one subapical pair, sometimes very close to apex, parallel or slightly diverging, 2(3) lateral pairs and one basal pair; apart from this often with a tiny pre-basal seta at least present on one side; 2–4 strong suberect preapical discal setae, together with some setulae forming a row in front of the marginal setae, the strongest pair in the middle sometimes subequal to the lateral setae, mixed with numerous tiny setulae.

***Legs* (Fig. 1)**: Claws and pulvilli on fore legs equal to or slightly longer than tarsal segment 5, the latter ca. 2 × as long as tarsal segment 4. Chaetotaxy variable and it is sometimes difficult to distinguish between seta and setula (seta here accepted if its length is subequal with the width of tibia at middle): Fore tibia with 4–7 anterodorsal setae, 2–5 posterodorsal setae in an irregular row and two or three posterior setae; preapical anterodorsal seta well developed, subequal with the preapical dorsal and preapical posterior setae.

***Wing* (Fig. 1)**: Wing veins apart from costal vein and vein R_4+5_ bare. Usually two costal spines (rarely one or three), the strongest lower spine 1.5–3 × as long as the surrounding costal setulae. Fourth and fifth costal section 1.8–2.4 (n = 6) × as long as sixth costal sector. Vein R_4+5_ at node with ventral and dorsal setulae. Bend of vein M acute, length of appendix 0.3–0.5 × the shortest distance from bend to wing edge. Cell r_4+5_ open at wing edge or rarely almost closed (according to Zimin normally short-stalked or closed in *G.mesnili*). Apical section of vein M concave reaching wing margin in front of wingtip.

***Abdomen* (Figs 1, 4)**: Domed, ground-vestiture erect or at least semierect on tergites 3 and 4, also ventrally. Syntergite 1+2 with middorsal depression reaching to the end of the segment. Chaetotaxy variable: Tergite 2 without a pair of median marginal setae, with 2(1)–3 lateral setae on each side. Tergite 3 with 2–4 pairs of unequally often irregularly set strong median discal setae, with a pair of median marginal setae and (1)2–3 lateral setae on each side. Tergite 4 with 2–3(4) pairs of unequally and likewise irregularly set strong median discal setae, in dorsal view with a full row of 10–14 marginal setae. Tergites 3 and 4 frequently with one median discal seta missing or set more laterally. Tergite 5 with two or three irregular rows of unequally strong discal setae and a row of medium strong marginal setae.

***Male terminalia* (Figs 1E, 4, 5)**: Tergite 6 completely reduced. Sternite 6 strongly asymmetrical, with the broader left arm articulating with segment 7+8. Segment 7+8 large and convex, slightly larger than epandrium, separated from tergite 5 by a wide membrane. Epandrium in profile with a subquadrate appearance, with numerous setulae (the longest placed dorsally). Sternite 5 (Fig. 1E, 4E) in ventral view with distinct posterolateral lobes and a broad medial cleft that anteriorly narrows to a small U-shaped indentation; length of cleft (measured from the tip of the posterolateral lobes, including the U-shaped cleft) 1.8–2.5 × its width (measured at the anteriormost incision). Lobes with widespread setulae, rounded or triangular at apex; along the inner edge towards the cleft distinctly concave, from inner dorsomedial surface showing a long narrow or backwards expanding process; U-shaped median incision in posterior medioventral region with a pair of densely spinulose wart-like lobes; basal plate bare (without setulae) ca. 0.40–0.45 × the length of the sternite itself, and 2.2–2.7 × as wide as long, anterior margin distinctly concave; transversal membranous stripe wide and narrow, almost touching the U-shaped indentation. Tergite 6 completely reduced. Sternite 6 strongly asymmetrical, with the broader left arm articulating with syntergosternite 7+8, the latter large and convex slightly larger than epandrium. Epandrium arched trough-shaped, in lateral view with a subtriangular appearance, with numerous setulae (the longest placed dorsally). Syncercus in lateral view (Fig. 5A) smoothly curved at apex; in caudal view (Fig. 5B) triangular, narrowed to a blunt or rounded apex, with long basal lobes; 1.7–2.1 × as long as maximal width (length measured from base of membranous median suture to apex), slightly incurved at middle and close to apex; numerous black setae/setulae laterally give *G.lucida* a characteristic appearance. Surstylus in lateral view (Fig. 5A) slightly curved (*G.lucida*) or arch-like bent (*G.magna*, *G.viridis*, and *G.zhelochovtsevi*), at least proximally along posterior margin with some tiny setulae, otherwise with numerous spread sensory pores; separated from epandrium by a very narrow membranous succession. Pregonite lance-like in caudal view; lobe-like in lateral view (Fig. 5C) with a hook-like projection apically and some sensorial hairs along its posterior margin. Postgonite quite narrow, broadly membranous apically and therefore with a hook-like appearance.

Aedeagus: Basiphallus with a basal projection and a distinct digitiform epiphallus. Distiphallus in lateral view (Fig. 5C) with the dorsal sclerite as two narrow separate arms extending from apex of basiphallus, close to the posterior edge of the distiphallus all the way to the anterior tip; i.e., not fused dorsomedial and consequently without a median projection; basis of ventral sclerite prominent, lateroventral region with a row of numerous spines; medioventral ridge in lateral view narrow and weakly sclerotised, in ventral view resembling X, with base directed towards the ventral sclerite and apex to the end of the dorsal sclerite.

**Figure 6.**
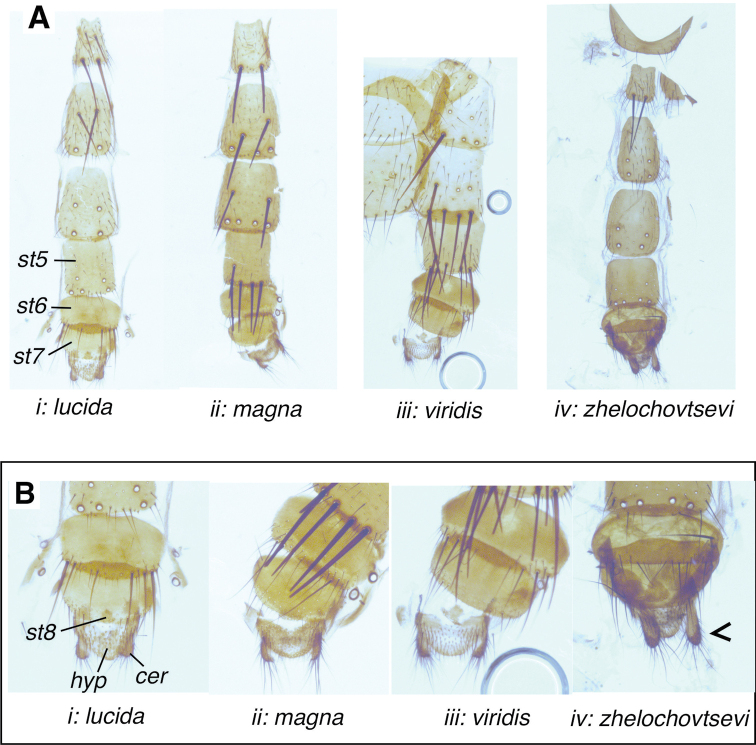
Comparison of *Gymnocheta* spp. female sternites and terminalia **A** ventral view of the sternites and terminalia of **i***G.lucida***ii***G.magna***iii***G.viridis* and **iv***G.zhelochovtsevi***B** detail of the female terminalia of **i***G.lucida***ii***G.magna***iii***G.viridis* and **iv***G.zhelochovtsevi*. Note the long cerci in *G.zhelochovtsevi*. All photographs by J. Pohjoismäki.

**Female** (Figs 3, 6): Differs from male as follows:

***Colouration***: Fronto-orbital plate with a more widespread metallic green colour; the metallic ground colour continues down on the upper part of parafacial; in large parts covered by greyish microtomentum in *G.zhelochovtsevi*.

***Head* (Fig. 3)**: Frons wider, at its narrowest point 0.8–1.0 × as wide as an eye in dorsal view. Frontal vitta either progressively tapering towards ocellar tubercle and at this level mostly narrower than width of fronto-orbital plate (*G.magna* and *G.viridis*), narrowest close to middle (*G.lucida*) or with almost parallel edges in anterior 1/2 or more and the narrowing (*G.zhelochovtsevi*). Fronto-orbital plate normally with fewer, 6–11 moderately strong medioclinate frontal setae. Ocellar tubercle with a pair of strong proclinate ocellar setae as in male but with shorter and more sparsely set setulae. Fronto-orbital plate with 2(3) strong proclinate orbital setae, the anterior one stronger, one upper, slightly lateroclinate, orbital seta at level of anterior ocelli. Inner vertical setae strong and crossed. Outer vertical setae strong and at least 0.6 × the length of the inner vertical setae. Postocular setae short and insignificantly bending forward. First flagellomere 1.3–1.8 × as long as pedicel.

***Legs***: Legs black. Claws and pulvilli on fore legs shorter ca. 0.7–0.8 × as long as tarsal segment 5, the latter 1.5–1.7 × longer than tarsal segment 4.

***Abdomen***: Tergite 5 trapezoid, along anterior margin ca. 2 × as wide as long.

***Female terminalia* (Fig. 6)**: Ovipositor short, deeply drawn into the lumen of the sternite 5. Uterus present, 3 equally sized spermathecae. Species are ovolarviparous; dissected females of *G.lucida* and *G.viridis* sometimes contained > 200 first instar larvae in different state of development. The first instar larvae are known to search actively for the host.

Tergite 6 reduced into narrow and somewhat elongated hemitergites, widely separated, in the membranous region accompanied by 0–3 tiny setulae posterior to the seventh spiracle and sometimes also one or two setulae in a more apical position, sixth spiracle in lateral margin of tergite 6. Sternite 6 shorter 0.5–0.7 × as long as, but slightly wider (1.1–1.2 ×) than sternite 5, 1.5–2 × as wide as long with 4–8 strong setae along the posterior margin accompanied by numerous setulae in posterior 1/5. Tergite 7 strongly reduced, divided into two widely separated small hemitergites located close to the lingulae, with 0–3 setulae in the adjacent membranous area, left and right seventh spiracles displaced in membrane between tergites 6 and 7, sometimes close to sixth spiracles. Sternite 7 ca. as long but slightly narrower than sternite 6, with numerous tiny setulae most of them restricted to the posterior margin, anterior 4/5 bare. Tergite 8 missing (fully reduced). Sternite 8 strongly reduced, hidden between hypoproct and sternite 7; covered by sensory pores (sensilla trichodea). Hypoproct in ventral view semi-circular to almost triangular, with a middorsal depression, densely covered with setulae, in lateral view (Fig. 6) with dorsal side straight or slightly bent towards the cerci, lingulae present. Epiproct strongly reduced (no sclerotising visible) but in caudal view indicated by the presence of 3–10 setulae inserted above the cerci. Cerci with numerous setulae of varying length, in lateral view the strongest setulae at least subequal in length with sternite 7.

The European species of *Gymnocheta* are treated in alphabetical order. All Finnish records are stored in the Finnish species database, www.laji.fi.

### Gymnocheta lucida

Taxon classificationAnimaliaDipteraTachinidae

Zimin

6F759372-8A63-5C27-AB09-248252BE1220

Gymnocheta lucida Zimin, 1958: 60. Lectotype ♂ [ZIN], by designation of Richter (1981: 917) (see below).

#### Material.

**Russia: *Lectotype*** ♂, by designation of Richter (1981: 917). Label: Майхэ близ Шкотова, Уссур. кр., 5.VI.1927, Штакельберг (Mayhe near Shkotov, Ussur. kr., 5.VI.1927, Stackelberg) – [Russia, Primorsky Krai, Ussuri district] [ZIN]. Examined from high quality photographs. **Japan**: (2♂♂, 1♀) 1♂: Honshu / 3. V. 1967 / H. Kurahashi // *Gymnochaeta* / *lucida* Mesn. (handwritten) / det. H. Shima, 1982 [BLKU]; 1♀: Honshu / 3. V. 1967 / H. Kurahashi [BLKU]; 1♂: [Aomori, Japan] / Inekari River, Koguriyama, / Hirosaki City / May 28, 2013 / D. Katô leg. // *Gymnocheta* / *viridis* Fall. ? (handwritten) / det. T. Tachi 2020 [BLKU]. **Finland**: (5♂♂, 5♀♀) 1♂: Finland, Tavastia australis, Tammela, Torronsuo, 60.74, 23.58, 13.vi.2004, K. Mattila leg. (BOLD Sample-ID JP00605, GenBank accession number KX843924) [TMNH]; 1♂: Finland, Karelia borealis, Ilomantsi, Pirhunvaara, 62.973; 31.406, 24.vi.2008, J. Kahanpää leg. (BOLD Sample-ID jka08-00018, GenBank accession number KX844119); 1♂: Finland, Ostrobottnia borealis borealis, Rovaniemi, Kivalo, 66.325; 26.854, 13.–25.vi.2014, I. Gonzales leg. [JPC]; 1♀: Finland, Lapponia Kemensis pars occidentalis, Kittilä, Vuotsonperän jänkä, 67.617; 25.45, 24.vii.2007, J. Salmela leg. (BOLD Sample-ID JP00603, GenBank accession number KX843771) [TMNH]; 1♀: Finland, Tavastia australis, Juupajoki, Lakkasuo, 61.798; 24.317, 12.vii.2007, J. Pohjoismäki leg. (BOLD Sample-ID JP00604, GenBank accession number KX843828) [TMNH]; 1♂: Finland, Karelia borealis, Lieksa, Lahnasuo, 63.118; 30.6144, 20.v.–11.vi.2015, J. Pohjoismäki leg. (BOLD Sample-ID JP2019005) [JPC]; 1♂: ibid. Dissected. [JPC]; 2♀♀: Finland, Karelia borealis, Lieksa, Lahnasuo, 63.118; 30.6144, 12.vi.–3.vii.2015, J. Pohjoismäki leg. [JPC]; 1♀: Finland, Karelia borealis, Polvijärvi, Tiaissuo, 62.950233; 29.424098, 7.–27.vii.2014, J. Pohjoismäki leg. [JPC]. For the boundaries of the Finnish geographical provinces see https://laji.fi/theme/emk). **Sweden**: (64♂♂, 33♀♀) 1♀: Lu. Lpm, Jokkmokk, Kallkällmyran, O1677655 N7384113, 18.vi.1978, leg. R.&T-B. Engelmark [REC]; 1♀: Lu. Lpm, Jokkmokk, Kallkällmyran, O1677655 N7384113, 22.vi.1978, leg. R.&T-B. Engelmark [REC]; 1♀: Lu. Lpm, Jokkmokk, Kallkällmyran, O1677655 N7384113, 27.vi.1978, leg. R.&T-B. Engelmark [REC]; 1♀: Lu. Lpm, Jokkmokk, Kallkällmyran, O1677655 N7384113, 02.vii.1978, leg. R.&T-B. Engelmark [REC]; 1♂: Lu. Lpm, Jokkmokk, Kallkällmyran, O1677655 N7384113, 06.vii.1978, leg. R.&T-B. Engelmark [REC]–1♀: Lu. Lpm, Jokkmokk, Kallkällmyran, O1677655 N7384113, 02.vii.1979, leg. R.&T-B. Engelmark [REC]; 1♂: Lu. Lpm, Jokkmokk, Kallkällmyran, O1677655 N7384113, 01.vi.1981, leg. R.&T-B. Engelmark [REC]; 1♀: Lu. Lpm, Jokkmokk, Keutatjape, O1667053 N7383578, 18.vi.1981, leg. R.&T-B. Engelmark [REC]; 1♂: Lu. Lpm, Jokkmokk, Kallkällmyran, O1677655 N7384113, 29.vi.1981, leg. R.&T-B. Engelmark [REC]; 1♂: Lu. Lpm, Jokkmokk, Kallkällmyran, O1677655 N7384113, 30.vi.1981, leg. R.&T-B. Engelmark [REC] Dissected.; 1♂: Lu. Lpm, Jokkmokk, Sasnekape, O1677032 N7380533, 13.vii.1981, leg. R.&T-B. Engelmark [REC]; 1♀: Lu. Lpm, Jokkmokk, Vaimat, ”Stormyren”, O1664900 N7384200, 16.vii.2008, leg. C. Bergström [CBC]; 1♀: Hls, Nordanstig, Ilsbo, Sörängsberget, O1564037 N6863588, 17.vii.1987, leg. C. Bergström [CBC]; 1♀: SE, Vrm, Kristinehamn, Flymossen, O1404043 N6580966, 14.vii.1985, leg. C. Bergström [CBC]; 4♂♂: SE, UP, Huddunge sn. Evighetsmossen, O1572552 N6657235, 30.v.1999, leg. C. Bergström [CBC], 1♂ dissected; 2♂♂: SE, UP, Huddunge sn. Evighetsmossen, O1572552 N6657235, 03.vi.1999, leg. C. Bergström [CBC]; 2♀♀: SE, UP, Huddunge sn. Evighetsmossen, O1572550 N6657230, 16.vi.2003, leg. C. Bergström [CBC] Dissected; 1♂: 30 / 220 // SE, UP, Huddunge sn. Evighetsmossen, O1572552 N6657235, 29.v.2020, leg. C. Bergström [CBC]; 2♂♂: 61–62 / 2020 // SE, UP, Huddunge sn. Evighetsmossen, O1572552 N6657235, 04.vi.2020, leg. C. Bergström [NHRS]; 2♂♂: 70–71 / 2020// SE, UP, Huddunge sn. Evighetsmossen, O1572552 N6657235, 05.vi.2020, leg. C. Bergström [CBC]; 3♂♂, 2♀♀: 72–76 / 2020 // SE, UP, Huddunge sn. Öjemossarna, O1571242 N6657364, 09.vi.2020, leg. C. Bergström [CBC]; 1♂: 82 / 2020 // SE, UP, Huddunge sn. Öjemossarna, O1571242 N6657364, 10.vi.2020, leg. C. Bergström [BLKU]; 1♂: 83–92 / 2020 // SE, UP, Huddunge sn. Öjemossarna, O1571242 N6657364, 10.vi.2020, leg. C. Bergström [CBC]; 6♂♂: 111–116 / 2020 // SE, UP, Huddunge sn. Evighetsmossen, O1572270 N6657616, 15.vi.2020, leg. C. Bergström [CBC]; 15♂♂: 117–131 / 2020 // SE, UP, Järlåsa sn. Ramsmossen, O1574877 N6649254, 18.vi.2020, leg. C. Bergström [CBC]; 2♀♀: 138–139 / 2020 // SE, UP, Huddunge sn. Evighetsmossen, O1572270 N6657616, 21.vi.2020, leg. C. Bergström [CBC]; 1♀: 141–142 / 2020 // SE, UP, Huddunge sn. Evighetsmossen, O1572270 N6657616, 21.vi.2020, leg. C. Bergström [BLKU]; 2♀♀: 141–142 / 2020 // SE, UP, Huddunge sn. Evighetsmossen, O1572270 N6657616, 21.vi.2020, leg. C. Bergström [NHRS]; 1♂♀: in copula, 9♂♂: 144–145, 154–162 / 2020 // SE, UP, Huddunge sn. Evighetsmossen, O1572552 N6657235, 21.vi.2020, leg. C. Bergström [CBC]; 1♂♀: in copula, 2♂♂,10♀♀: 183, 165-175,184-185 / 2020 // SE, UP, Huddunge sn. Evighetsmossen, O1572552 N6657235, 23.vi.2020, leg. C. Bergström [CBC]; 1♂, 2♀♀: 194–195, 200 / 2020 // SE, UP, Huddunge sn. Evighetsmossen, O1572552 N6657235, 29.vi.2020, leg. C. Bergström [CBC]

#### Diagnosis.

*Gymnochetalucida* Zimin is a dark metallic olive to bronze-green tachinid, often with a matt appearance due to a dense microtomentum, which also gives the genal dilation a greyish white appearance.

#### Redescription.

Body length: 7.2–9.5 mm (n = 29).

**Male** (Figs 2A, 4A, Ei, 5A:i, B:i, C:i).

***Colouration***: Head covered with dense greyish white microtomentum. The metallic ground colour of the genal dilation shines through weakly, compared to *G.magna* and *G.viridis*. However, this difference is sometimes hard to recognise, as the interpretation depends on the direction of the incidence of light, and the specimens should be viewed from different angles. Facial plate black normally without a metallic green spot. Occiput, postgena, genal dilation dark metallic bronze-green in ground colour, frontal plate sometimes narrowly metallic bronze-green along the frontal setae, ocellar triangle mostly black. Palpus deeply black (charcoal) or dark brown in older bleached specimens. Prementum black, labella brown. Thorax and abdomen metallic dark olive green in ground colour, shine depending on the direction of the incidence of light; covered with greyish white microtomentum. The intensity of the microtomentum varies, some specimens having more matt appearance than others and in general the microtomentum in males is more dominant than in females. The intensity of the green colouration is variable, some specimens are more lucid green while others, especially from cooler locations, are very dark, almost blackish green. Scutum, when viewed from the side and slightly from behind, with four longitudinal stripes of microtomentum, changing from grey to purple depending on the direction of the incident light. In aged specimens, caught late in the season, the microtomentum can be worn out and give the specimens a polished appearance for the most dorsal part of scutum and postpronotum. Proepisternum black and densely covered with microtomentum. Fore coxa in anterodorsal region and sometimes also femora with remnants of metallic shine, covered with light grey microtomentum. Wing membrane around crossvein r-m narrowly (sometimes somewhat indistinctly) infuscate. Tegula and basicosta black.

***Head* (Fig. 2A)**: Frons at its narrowest point, 0.43–0.51 (n = 19) × as wide as an eye in dorsal view. Frontal vitta tapering toward middle (sometimes narrowest at this point) and then parallel-sided towards the ocellar tubercle. Head in profile somewhat protruding at level of antennal insertion, width of parafacial at this level ca. 0.55–0.65 × (n = 17) the horizontal eye diameter. Fronto-orbital plate with a row of 10–12 medioclinate moderately strong frontal setae and some additional setulae, uppermost setula tiny sometimes slightly reclinate, four or five setae descending on upper part of parafacial, reaching the middle of the pedicel with the row curving laterally, and here sometimes attended by some setulae; fronto-orbital plate outside the frontal row of setae with sparsely, spread, short and tiny setulae. Height of face slightly shorter than the length of frons (n = 17). Gena in profile at narrowest point, 0.29–0.38 (n = 14) × as high as vertical eye diameter. Vibrissa normally well-developed but in some specimens there is no distinct vibrissa but two or three equally strong supravibrissal setae. Facial plate slightly convex but hardly visible in profile, but lower facial margin protruding especially in the middle but in profile hardly below the subvibrissal setae. Facial ridge on less than lower 1/5 with 2–4 strong and 0–2 additional thinner supravibrissal setae (length of them at least equals narrowest width of parafacial) and 2–4 thin and short setulae. Ridge below vibrissa with 3–5 strong subvibrissal setae continuous with the genal setae, longest ca. 0.5 × the length of vibrissa. Inner vertical setae strong and crossed, longer than the ocellar setae, outer vertical setae less developed, subequal with the adjacent postocular setae. Postocular setae long and thin, apically pronouncedly bending forward over the eyes. Occiput with a pair of postocellar setae mostly subequal with the outer vertical setae, but sometimes missing. Palpus slightly clavate at tip, subequal to the length of the antenna and densely covered with short black setulae 2 strong preapical setulae and four or five irregular ventral setulae. Antenna: First flagellomere in profile 1.10–1.32 ×) (n = 15) wider than parafacial at narrowest point, and 1.39–1.56 (n = 15) × as long as pedicel. Arista widened in at least its basal 1/2 (sometimes almost in basal 2/3) and gradually tapering to apex.

***Thorax***: Prosternum bare. Scutum with 3(2)+3 acrostichal, 3(2)+4 dorsocentral and 1+3 intra-alar setae. Ground vestiture on scutum (consisting of thin setulae) dense and erect, longest setulae subequal to the shortest setae. Scutellum normally with four (rarely five) pairs of strong marginal setae, the subapical pair sometimes inserted close to apex, a tiny pre-basal seta present at least on one side; normally four suberect preapical discal setae, forming a row in front of the marginal setae, the strongest pair in the middle sometimes subequal to the lateral setae, mixed with numerous tiny setulae the longest at least 1/5 as long as the strong preapical setae.

***Legs***: Claws and pulvilli on fore legs ca. 1.1–1.2 (n = 5) × as long as tarsal segment 5, the latter 2.0–2.1 (n = 5) × as long as tarsal segment 4. Fore tibia with a row of 4–6 anterodorsal setae and two or three thin setulae, 4–7 setae/setulae in an irregularly posterior or posterodorsal position (often two or three of them representing setae but sometimes only five tiny setulae present): preapical anterodorsal seta well developed, subequal with the preapical dorsal and preapical posterior setae. Mid tibia with a row of 4–6 anterodorsal setae the strongest in the middle, 2–4 posterodorsal setae (at least two are strong), two posterior setae, one ventral seta often accompanied by a tiny setula. Hind tibia with equally long preapical anterodorsal and posterodorsal setae, apical posteroventral seta ca. 1/2 length of the anterioventral seta; a continuous irregular row of 8–11 anterodorsal setae / setulae of which 4–6 represent strong setae, three or four posterodorsal setae and two or three anteroventral setae.

***Wing***: Usually two costal spines (rarely one or three), the strongest lower spine ca. 3 × as long as the surrounding costal setulae, normally only somewhat shorter than crossvein r-m. Fourth and fifth costal section 1.8–2.1 (n = 6) × as long as sixth costal sector. Vein R_4+5_ with 3–7 ventral and 2–9 dorsal setulae (in one deviating specimen with nine setulae almost reaching r-m). Cell r_4+5_ often somewhat narrowly open at wing edge, 0.50–0.75 × the length of crossvein r-m.

***Abdomen* (Fig. 4A)**: Domed, ground-vestiture erect or at least semierect on tergites 3 and 4, also ventrally. Tergite 2 with two (rarely one) lateral setae on each side. Tergite 3 with two or three pairs of unequally often irregularly set strong median discal setae, with a pair of median marginal setae and 2(3) lateral setae on each side. Tergite 4 with two or three pairs of unequally and likewise irregularly set strong median discal setae, in dorsal view with a full row of 10–12 marginal setae. Tergites 3 and 4 frequently with one median discal seta missing or set more laterally. Tergite 5 with two or three irregular rows of unequally strong discal setae and a row of medium strong marginal setae.

***Terminalia*** (4 dissections) (Figs 4A, Ei, 5A:i, B:i, C:i): Sternite 5 (Fig. 4A, Ei) in ventral view with finger-like lobes; length of cleft (measured from the anteriormost indentation) 2.5 × its width. Lobes with widespread setulae, rounded at apex. Dorsomedial process narrow and somewhat indistinct. Basal plate bare (without setulae) ca. 0.4 × as long as sternite 5 and 2.2–2.3 × as wide as long. Syncercus in profile (Fig. 5A:i) smoothly curved before apex, in caudal view (Fig. 5B:i) rounded at apex; 2 × as long as its maximal width (measured from base of membranous median suture to apex); vaguely curved inwards at middle and close to apex; numerous dorsolateral setulae at middle gives a characteristic dense and fur-like appearance. Surstylus (Fig. 5A:i) bacilliform with some tiny setulae most prominent along posterior margin in the proximal region, otherwise with numerous spread sensory pores, straight in caudal view, in profile gradually tapering at base, smoothly curved, evenly thick apart from an indicated widening at apex, bent towards syncercus. Pregonite lance-like in caudal view; lobe-like in profile (Fig. 5C:i) with a narrow hook-like projection apically, shortly tapering at apex, with short sensorial hairs along its posterior margin, anterior margin to some extent incurved. Aedeagus: distiphallus in profile compact and widest close to apex (Fig. 5C:i).

**Female** (Figs 3A, 6A:i, B:i): Differs from male as follows:

***Colouration***: Fronto-orbital plate almost entirely metallic bronze-green when viewed from behind and slightly from above; the metallic ground colour at least partly interrupted on the upper part of parafacial, sometimes in patches reaching the level of the lowermost frontal setae. However, most of the metallic ground colour on upper part of parafacial and anterior 1/3 of fronto-orbital plate is covered by greyish white microtomentum, dense especially along eye margin and frontal setae, but when viewed from side and slightly from above with golden reflections. Thorax and abdomen dark metallic green to bronze-green, microtomentum normally thinner than in males, most intense on the pleura and episternum.

***Head* (Fig. 3A)**: Frons wider, at its narrowest point 0.78–0.93 (n = 10) × as wide as an eye in dorsal view. Frontal vitta tapering toward middle (sometimes narrowest at this point) and then gradually widening towards the ocellar tubercle, its width at ocellar tubercle exceeding the width of fronto-orbital plate at this point. Fronto-orbital plate normally with fewer, 7–11 moderately strong medioclinate frontal setae. Outer vertical setae strong 0.65–0.75 × the length of inner vertical setae; subequal with the ocellar setae and the posterior proclinate orbital setae, distinctly stronger than the lateroclinate orbital setae and at least twice the length of the adjacent postocular setae. Postocellar setae short, tiny and subequal with the upper postocular setae. First flagellomere 1.29–1.46 (n = 10) × as long as pedicel, normally ca. as wide as parafacial at narrowest point.

***Legs***: Claws and pulvilli on fore legs shorter, ca. 0.7–0.8 (n = 5) × as long as tarsal segment 5, the latter 1.5–1.6 (n = 5) × as long as tarsal segment 4.

***Abdomen***: Ground vestiture dorsolateral on tergites 3 and 4 distinctly prone contrasting to the erected setulae between the median discal setae. Tergites 3 and 4 with two or three pairs of median discal setae. Sternite 5 somewhat elongated, 1.1–1.2 × as long as its maximal width (Fig. 6A:i), with 5–8 strong setae in posterior 1/2, three or four of them along the posterior margin, in posterior 2/3 accompanied by numerous (50–60) irregularly spread setulae of varying size.

***Terminalia*** (2 dissections) (Fig. 6A:i, B:i): Tergite 6 divided into narrow and somewhat elongated hemitergites, widely separated, each closely accompanied by 0–3 tiny setulae in the posterior membranous area (and sometimes indicated by a pair of setulae in a more apical position). Sternite 6 shorter 0.6–0.7 × as long as but slightly wider (1.2 ×) than sternite 5, 1.5–1.6 × as wide as long with 8 setae (n = 3) along the posterior margin and with numerous tiny setulae in posterior 1/5. Tergite 7 with 0–3 setulae in the adjacent membranous area. Sternite 8 with 6–8 sensory pores (sensilla trichodea). Hypoproct (Fig. 6B:i) in ventral view almost triangular, apex only slightly rounded (studied in ventral and somewhat caudal position pointed at apex): with a quite poorly developed medioventral depression, densely covered with setulae that laterally are longer; in profile slightly bent towards the cerci, apex of hypoproct hardly reaching tip of cerci, lingulae well developed. Epiproct in caudal view indicated by the presence of three or four setulae inserted above the cerci. Cerci short with numerous setulae of varying length, the strongest setulae in profile subequal to the length of sternite 7.

#### DNA.

**Figure 7.**
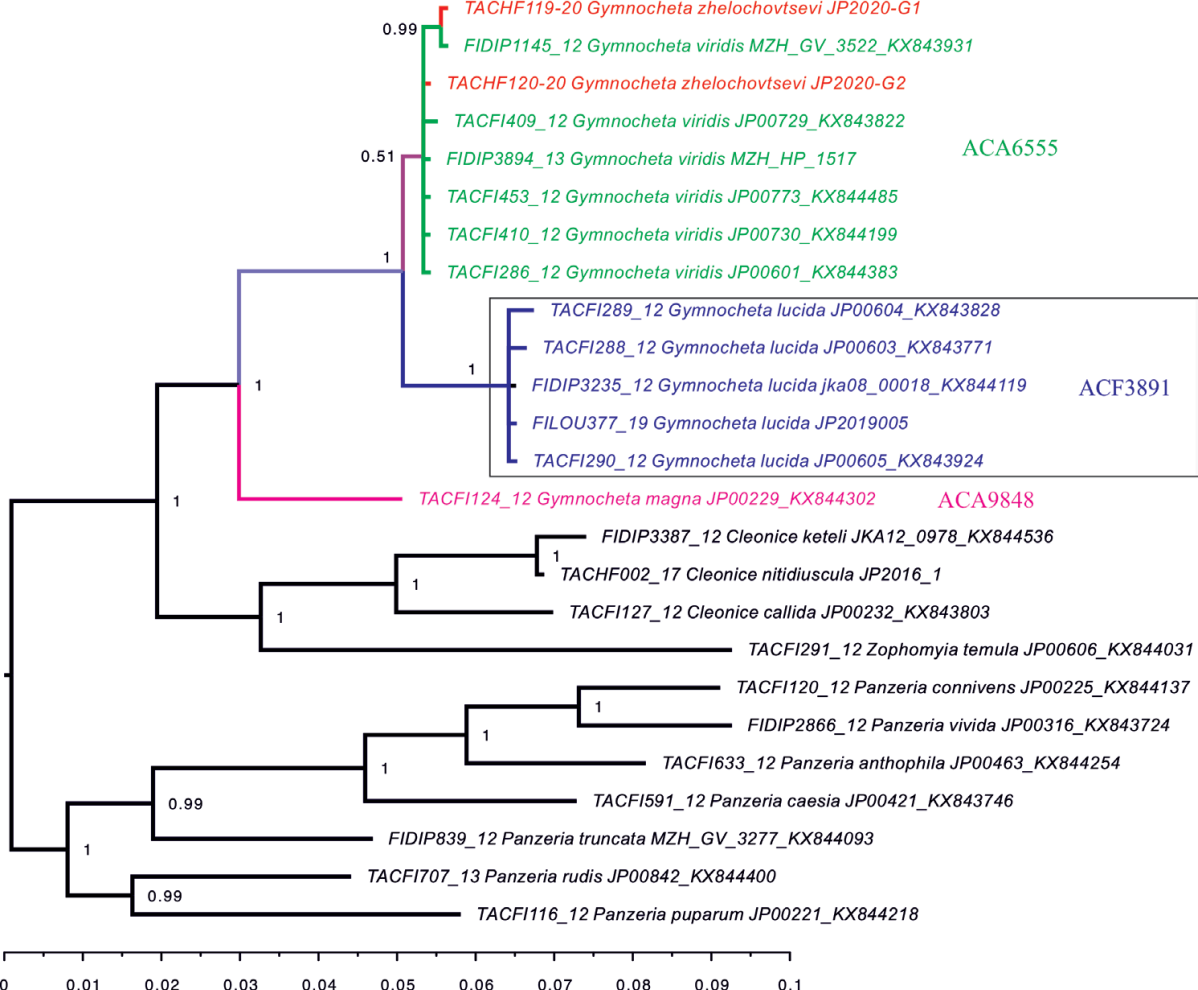
Maximum likelihood tree of the *Co1* sequences of the Nordic *Gymnocheta* spp. and related Ernestiini. Numbers at nodes indicate posterior probabilities and scale bar the relative sequence divergence. BOLD progress ID given before the species name and sample ID as well as GenBank access number given after it. The DNA barcodes do not differ between the Nordic *G.zhelochovtsevi* and *G.viridis* (BOLD:ACA6555) but differ significantly from *G.lucida* (BOLD:ACF3891) and *G.magna* (BOLD:ACA9848).

The Co1 DNA barcode sequence of *G.lucida* differs markedly from the other European species of *Gymnocheta* (Fig. 7). It has been assigned a species-specific Barcode Index Number (BIN): BOLD:ACF3891.

#### Distribution.

*Gymnochetalucida* was previously known only from the Russian Far East and Japan (Richter 2004) and we report it here for the first time as also common and widespread in Finland and northern Sweden. In the Barcode of Life Database (BOLD), there is one match for the species-specific BIN from Norway. This species likely occurs also elsewhere in Europe but has been confused with the common *G.viridis*, and may have a continuous range throughout the Central Palaearctic.

#### Biology.

Almost all Finnish records of *G.lucida* are from ombrotrophic raised bogs. The only exception is the record from Rovaniemi, Lapland (Finland), collected from a pine forest habitat. However, it is typical that insect species that are specific to bogs or moorlands in the south extend their habitat preferences to open forests or heathlands in the north. *Gymnochetaviridis* is also sometimes recorded from bogs, so the habitat is not a reliable indicator for the species. Similarly, the habitats in Uppland are best described as ombrotrophic bogs separated by different types of coniferous woodlands. The bogs partly covered with dwarfed pine trees, and a scrub layer dominated by *Vacciniumuliginosum*, *Rhododendrontomentosum*, *Myricagale*, *Callunavulgaris*, and *Betulanana*. The coniferous area surrounding the bogs consists discontinuously of rocky outcrops densely covered by different lichens, i.e., *Cladonia* sp. and *Cetrariaislandica*, and old pine trees, and sinks also with old pine trees and single spruces; the scrub layer here is dominated by *Vacciniummyrtillus* but also with elements of *Vacciniumvitis-idaea*, *Rhododendrontomentosum*, and *Vacciniumuliginosum*. The three collection sites of Kallkällmyran, Keutatjape, and Sasnnekape are located just south of the Arctic Circle in Jokkmokk, Lule Lappmark (Sweden). They have been described as rich to medium rich fens in the coniferous zone near Jokkmokk (Engelmark and Engelmark 1989). These minerotrophic fens are fed with ferriferous groundwater and the complex vegetation is characterised by *Saxifragahirculus*.

Males appear in late May and individuals are first and foremost observed in small sunlit clearings in the sinks between the rocky outcrops, sitting on leaves and twigs of blueberries apparently watching for approaching females. Some of these clearings likely represent hot spots for aggregation. These are frequently attended by males that fly out sporadically from time to time in response to another passing male, but in one of these hot spots mating was observed on two occasions. One male was collected when visiting the flowers of *Rhododendrontomentosum*. Females appear around second week of June and they are, apart from the ones observed *in copula*, found on the edge of the bogs close to the rocky outcrops. They have been observed between late June to mid-July in characteristic slow, searching flight, low in the vegetation often just a couple of inches above the moss layer, examining tufts of sedges and shrubs, such as *Vacciniumuliginosum* and *Betulanana*. The hosts are currently unknown but concealed larvae of Noctuidae or Erebiidae (Lepidoptera), living in the habitats described above, are most likely.

### Gymnocheta magna

Taxon classificationAnimaliaDipteraTachinidae

Zimin

B16C638F-BF20-5A5B-BC59-D94E10662CE3

Gymnocheta magna Zimin, 1958: 53. Lectotype ♂ [ZIN], by designation of Richter (1981: 917) (see below).

#### Material.

**Russia: *Lectotype*** ♂, by designation of Richter (1981: 917). Type locality: Ущ. Суцэуктз, ю.-а. Кентей, Монголия, конец V. 1924 (Козлов). Mongolia, Hentiy Aimag [as “Kentei” in Russian], Sutszukte. Not examined. **Sweden**: (4♂♂, 6♀♀) 1♀: SE, UP, Årsta/Slavsta, O1606484 N6639427, 16.vi.1980, leg. C. Bergström [CBC]; 2♀♀: SE, UP, Kvarnbo, O1598600 N6637685, 6.vi.1982, leg. C. Bergström [CBC]; 1♀: SE, UP, Ekebydalen, O1600268 N6637514, 6.vii.1983, leg. C. Bergström [CBC]; 1♂: SE, UP, Skogsängen, Nåsten, O6636152 N1597099, 11.vi.1986, leg. C. Bergström [CBC]; 2♂♂, 1♀: SE, UP, Skogsängen, Nåsten, O6636152 N1597099, 22.vi.1987, leg. C. Bergström [CBC]; 1♀: SE, UP, Skogsängen, Nåsten, O6636152 N1597099, 27.vi.1987, leg. C. Bergström [CBC]. **Finland**: (2♂♂, 3♀♀) 1♂: Ostrobottnia media, Raahe, Hakotauri, 64.7002; 24.4736, 3.vii.2006, K. Varpenius leg. (BOLD Sample-ID JP00229, GenBank accession number KX844302) [TMNH]–1♂: Finland, Karelia borealis, Ilomantsi, Ilajansuo, 62.915455; 31.201721, 20.vi.2013, J. Pohjoismäki leg. [JPC]; 1♀: Ostrobottnia kajanensis, Kuhmo, Ulvinsalo, 63.973506; 30.319950, 22.vi.2019, J. Pohjoismäki leg. [JPC]; 1♀: Regio Aboensis, Mietoinen, Perkko, 6733:3222, 9.v.-16.vi.2004, A. Haarto leg. [AHC]; 1♀: Tavastia australis: Pälkäne, 6807:3353, 27.vi.1993, Y. Ranta leg. [AHC]. **Japan**: (2♂♂, 1♀) 1♂: [KYUSHU] / Takachiho-mine / Kagoshima pref. // VI–7–1960 / H. Shima leg. (handwritten) // *Gymnochaetopsis* / *magna* Zimin / det. H. Shima 2020 [BLKU]; 1♂: [KYUSHU] / Mt. Takachiho / Kagoshima // 16.vi.1968 / A. Nakanishi (handwritten) // *Gymnochaetopsis* / *magna* Zimin / det. H. Shima 2020 [BLKU]; 1 ♀: [HONSHU] / Niigata / Myôkôkgen-sho / Sasagamine / 22.vi.1996 / T. Tachi [BLKU].

**Diagnosis.** A lucid green *Gymnocheta* species with a notably protruding lower facial margin and green femora. This is also the only European *Gymnocheta* species with short costal spines.

#### Redescription

[values in brackets refer to the holotype of *T.viridis* Fallén]. Body length: 7.8–12.1 [12.1] mm (n = 10).

**Male** (Figs 1B–E, 2B, 4Eii, 5A:ii, B:ii, C:ii).

***Colouration***: Head covered with a greyish white microtomentum. The metallic ground colour subshiny on the genal dilation but the shine/intensity is depending on the direction of the incident light. Facial plate with a greyish white microtomentum; lower protruding area however with a distinct mint metallic green tint. Occiput, postgena, genal dilation, ocellar tubercle metallic green in ground colour. Fronto-orbital plate narrowly but mostly distinctive mint metallic green along the frontal row of setae. Palpus, clypeus (sometimes with a metallic green tint) and prementum dark brown to black, labella brown beige. Thorax and abdomen bright mint metallic green in ground colour, contrasting with gilt or purple lustre depending on the direction of the incident light, with an indistinct greyish white microtomentum. Scutum when viewed from the side and slightly from behind with four longitudinal stipes changing from grey to gilt or purple depending on the direction of the incident light. Proepisternum normally with the metallic green ground colour subshiny, but less so in dwarfish specimens. Fore coxa in anterior region and femur posteriorly mostly with a metallic green shine. Wing membrane around crossvein r-m rarely infuscated. Tegula and basicosta dark brown or black.

***Head* (Figs 1C, 2B)**: Frons narrower than in *G.lucida*, at its narrowest point 0.31–0.38 [0.37] (n = 6) × as wide as an eye in dorsal view. Frontal vitta gradually tapering toward ocellar tubercle. Frons in lateral view somewhat protruding, width of parafacial at level of antennal insertion 0.59–0.72 [0.72] × (n = 6) the horizontal eye diameter. Fronto-orbital plate with a row of 10–14 [11] medioclinate frontal setae, mixed with some setulae, three to five setae descending on upper part of parafacial, reaching the middle of the pedicel with the row curving laterally, uppermost tiny setulae sometimes reclinate; orbital plate at level of ocellar tubercle with numerous moderately long and tiny setulae, frontal plate with more sparsely spread short and tiny setulae. Height of face 0.79–0.85 [0.80] × the length of frons (n = 6). Gena in profile at narrowest point, 0.35–0.39 [0.38] (n = 6) × as high as vertical eye diameter. Facial plate bulged medially and slightly visible in lateral view, lower facial margin distinctly protruding, in strict lateral view visible in front and below the vibrissa. Facial ridge on lower 0.20–0.25 with 1–3 [2] strong and 0–3 [2] additional thinner supravibrissal setae (length of them at least subequal to narrowest width of parafacial) and 2–5 thin and short setulae. Below the vibrissa 4–7 [7] strong subvibrissal setae continuous in front of the genal setae, the posteriormost subvibrissal seta close to the foremost lower genal margin. Inner vertical setae crossed, thin and weaker or at most subequal in length with the ocellar setae, outer vertical setae at most subequal with the adjacent postocular setae. Postocular setae long and thin, apically pronouncedly bending forward over the eyes. Occiput and postgena both with white to greyish white hairs. Antennae: First flagellomere in profile 0.90–1.06 (n = 5) × the actual width of parafacial; at narrowest point; 1.52–1.79 (n = 5) as long as than pedicel. Arista widened in its proximal ¼–1/3, gradually tapering towards apex.

***Thorax* (Fig. 1B)**: Prosternum frequently with tiny setulae [without]. Scutum with 3(4)+3 [3+3] acrostichal, 3–4 dorsocentral and 1+3 intra-alar setae. Scutellum with 4(5) [5] pairs of strong setae along margin, apical and subapical pairs slightly diverging; 2–4 [3] suberect dorsal scutellar setae, in front of the subapical setae, the strongest subequal to the lateral setae, rarely with an additional pair of medium strong median dorsal setae; tiny dorsal setulae numerous, the longest at least 1/2 length of the strongest dorsal seta.

***Legs***: Claws and pulvilli on fore legs slightly longer than fifth tarsal segment, the latter 1.7–2.0 (n = 3) × as long as the fourth. Fore tibia with a row of 5–6 anterodorsal setae, and an irregular row of 2–4 posterodorsal and 2 posterior setae; preapical anterodorsal seta subequal with the preapical dorsal seta, preapical posterodorsal seta short, its length rarely exceeding 1/3 of the dorsal seta. Mid tibia with three or four anterodorsal setae, the strongest in the middle of row, five or six posterior (pd and p) setae, one ventral seta often accompanied by an additional tiny setula. Hind tibia with a continuous row of nine or ten unequally strong anterodorsal setae / setulae of which four or five represent strong setae, three or four posterodorsal setae, the lowest one the strongest, and two or three anteroventral setae.

***Wing***: (Fig. 1B, 1D) Usually two costal spines, both short and the strongest ventral spine at most 1.5 × as long as the strongest surrounding costal setulae, normally only slightly exceeding 1/2 the length of crossvein r-m. CS_4_ + CS_5_ 2.0–2.3 (n = 3) × as long as CS_6_. Vein R_4+5_ at base with 2–4 ventral and 3–5 dorsal setulae.

***Abdomen* (Fig. 1B)**: Tergite 2 without a pair of median marginal setae, with 1(2) lateral marginal setae on each side. Tergites 3 and 4 with two or three unequally strong and often irregularly set pairs of median discal setae (sometimes two pairs and one unmatched seta). Tergite 3 with a pair of medial marginal setae, tergite 4 in dorsal view with a full row of 10–12 [10] marginal setae, tergite 5 with two or three irregular rows of strong and medium strong discal setae and a dense row of weak marginal setae.

***Terminalia*** (two dissections) (Figs 1E, 4Eii, 5A:ii, B:ii, C:ii): Sternite 5 (Figs 1E, 4Eii) in ventral view with short and wide lobes; length of cleft 1.7–1.8 × its maximal width. Lobes with widespread tiny setulae, curved inward at apex (posteriormost region in ventral and slightly lateral view with a subtriangular appearance); Dorsomedial process black and expanding backwards, strong, and very characteristic in part separated from sternite 5. Basal plate bare (without setulae) ca. 0.45 × as long as sternite 5 and 2.2–2.3 × as wide as long. Syncercus in lateral view (Fig. 5A:ii) smoothly curved at apex, similar to *G.lucida*; in caudal view (Fig. 5B:ii) somewhat resembling a pointed triangle with long basal lobes; 1.7–1.8 × as long as its maximal width (measured from base of membranous median suture to apex), slightly incurved at middle and close to a characteristic wide and blunt apex, dorsolateral at middle with numerous relatively long setulae. Surstylus (Fig. 5A:ii) slender, straight in caudal view; in lateral view slightly arch-like bent at ca. middle, almost evenly thick apart from apex that is slightly widening, bent towards syncercus. Pregonite lobe-like in lateral view with a wide hook-like projection, with short sensorial hairs along its proximal posterior margin, anterior margin slightly more curved than in *G.lucida*. Aedeagus: Distiphallus (Fig. 5C:ii) in lateral view compact but not widening close to apex.

**Female** (Figs 3B, 6A:ii, B:ii): Differs from male as follows:

***Colouration***: Fronto-orbital plate vivid mint metallic green when viewed from behind and slightly from above; the metallic ground colour continues down on the upper part of parafacial reaching the level of the lowermost frontal setae; the upper part of parafacial and anterior 1/3 of fronto-orbital plate with a thin greyish white microtomentum, visible along eye margin and as a slim line at the edge of frontal vitta. Thorax and abdomen vivid metallic green, at most with thin whitish microtomentum.

***Head* (Fig. 3B)**: Frons wider, at its narrowest point 0.75–0.83 (n = 5) × as wide as an eye in dorsal view. Frontal vitta gradually tapering towards ocellar tubercle, at this level often narrower than width of fronto-orbital plate. Fronto-orbital plate normally with fewer, 8–11 moderately strong medioclinate frontal setae. Outer vertical setae fairly stronger than the ocellar setae, shorter than the posteriormost proclinate orbital setae, at least twice the length of the adjacent postocular setae. Postocellar setae short and tiny and subequal with the upper postocular setae. One female with a long and slender first flagellomere otherwise not deviating from the situation in males 1.55–1.88) (n = 5) × as long as pedicel; its maximum width 0.94–1.08 (n = 5) × the actual width of parafacial at narrowest point.

***Legs***: Claws and pulvilli on fore legs shorter ca. 0.7–0.8 × as long as tarsal segment 5, the latter 1.5–1.7 × as long as tarsal segment 4.

***Abdomen***: Ground vestiture dorsolateral on tergites 3 and 4 distinctly prone contrasting to the erected setulae between the median discal setae. Tergites 3 and 4 normally with two or three pairs of median discal setae, tergites 3 and 4 occasionally devoid of one seta. Sternite 5 (Fig. 6A:ii) somewhat elongated, 1.1–1.2 × as long as its maximal width, with four strong setae along posterior margin accompanied by numerous irregularly spread setulae of varying size and one to two additional strong seta in posterior 4/5.

***Terminalia***: (two dissections) (Fig. 6B:ii)Tergite 6 divided into narrow and somewhat elongated hemitergites, widely separated, each in the membranous area accompanied by 3 tiny setulae posterior to the seventh spiracle and one or two setulae in a more apical position. Sternite 6 shorter, 0.5–0.6 × as long as but slightly wider (1.2 ×) than sternite 5, 2 × as wide as long with four setulae along the posterior margin and with numerous tiny setulae in posterior 1/5. Tergite 7 with three setulae in the adjacent membranous area close to lingulae. Sternite 8 strongly reduced, hidden between hypoproct and sternite 7; compared with *G.lucida* and *G.viridis* rather well developed; possesses at least 20 sensorial pores of which some are provided with tiny setulae; laterally on both sides with a long setula. Hypoproct (Fig. 6B:ii) in ventral view semicircle-shaped, with a distinct middorsal depression, densely covered with setulae, laterally on both sides with one longer setula, in lateral view not bent towards the cerci, apex of hypoproct reaching tip of cerci, lingulae well developed. Epiproct strongly reduced (no sclerotising visible) but in caudal view indicated by the presence of up to ten setulae inserted above the cerci. Cerci with numerous setulae of varying length, in lateral view the strongest setulae are at least subequal in length with sternite 7.

#### DNA.

*Gymnochetamagna* has a unique Co1 barcode sequence, BOLD:ACA9848 (Fig. 7).

#### Distribution.

Widely distributed in the Palaearctic Region (O´Hara et al. 2009). *Gymnochetamagna* is a relatively rare but widespread species in northern Europe. It was recorded as new to Sweden in Hedström (1985) and to Norway in Haraldseide (2012). It was listed from Finland in the checklist by Hackman (1980). The oldest specimen in MZH is from 1865, from Finland.

#### Biology.

The biology of *Gymnochetamagna* is poorly known. This is a summer species in the Nordic countries with a flight time from early June to mid-July. It is not a heathland specialist as suggested by some sparse records from Central Europe (Tschorsnig and Herting 1994), but has also been caught from meadows, forest margins and gardens (see also Fallén’s notes below). Both sexes visit flowers, especially those of cow parsnip, *Anthriscussylvestris* (L.) and caraway, *Carumcarvi* L. Females have been observed basking on aspen leaves. Hosts are unknown but are likely to be concealed larvae of Erebidae (Lepidoptera); see *G.viridis*.

### Gymnocheta viridis

Taxon classificationAnimaliaDipteraTachinidae

(Fallén)

77B7C000-5A50-5F11-91F9-C16A0E4393F6

#### Material.

***Holotype*** ♂ with a Fallén faded handwritten ink label reading (Fig. 1F): *Tachina* / *viridis* / ♂ Fallén. It also bears the following labels: Holotype ♂ / *Tachina* / *viridis* Fallén, 1810 / det. Bergström 2003 // *Gymnocheta* ♂ / *Tachina* (Fallén) / = *magna* Zimin SYN. N. / det. Bergström 2003. [NHRS, catalogue number NHRS-GULI000070873]. **Sweden**: (53♂♂, 16♀♀): ♂ (Fig. 1A): 3 / 2007 (red frame) // SE, UP, Uppsala, / Flogsta, Ekebydalen / O1600393 N6637642 / 2007-04-30/ leg. Christer Bergström // *Gymnocheta viridis* (Fallén) / J. Pohjoismäki & / C. Bergström 2021 [NHRS]. 3♂♂: SE, SÖ, Katrineholm, / Warbro, Hammarvik / O15405 N65458 1983-05-30 / leg. C. Bergström [CBC]; 5♂♂, 2♀♀: SE, SÖ, Katrineholm, / Warbro, Hammarvik / O15405 N65458 1985-05-25 / leg. C. Bergström [CBC]; 6♂♂: SE, SÖ, Katrineholm, / Warbro, Hammarvik / O15405 N65458 1985-05-26 / leg. C. Bergström [CBC]; 2♂♂: SE, SÖ, Katrineholm, / Warbro, Hammarvik / O15405 N65458 1985-05-27 / leg. C. Bergström [CBC]; 1♂, 3♀♀: SE, SÖ, Katrineholm, / Warbro, Hammarvik / O15405 N65458 1986-05-17 / leg. C. Bergström [CBC]; 1♀: SE, SÖ, Katrineholm, / Warbro, Hammarvik / O15405 N65458 1987-06-21 / leg. C. Bergström [CBC]; 1♀: SE, UP, Flogsta / Ekebydalen, / O16007 N66375 2000-06-15 / leg. C. Bergström [CBC]; 1♂: SE, UP Flogsta / Ekebydalen, / O16007 N66375 2000-05-11 / leg. C. Bergström [CBC]; 1♂: SE, UP, Enköping, / Fånö herrgård / O15890 N66069 2001-05-13 / leg. C. Bergström [CBC]; 5♂♂: SE, UP, Uppsala näs fg. / Sätrasjön / O1594026 N6634171 2001-05-10 / leg. C. Bergström [CBC]; 8♂♂: SE, UP, Enköping, / Fånö herrgård / O15890 N66069 2001-05-27 / leg. C. Bergström [CBC]; 2♂♂: UP, Uppsala näs fg. / Sätrasjön / O1594026 N6634171 2001-06-01 / leg. C. Bergström [CBC]; 1♂, 2♀♀: SE, UP, Uppsala näs fg. / Sätrasjön / O1594026 N6634171 2001-06-03 / leg. C. Bergström [CBC]; 3♂♂: 1381,1384, 1387 (red labels) // SE, UP, Uppsala näs fg. / Sätrasjön / O1594300 N6634400 2001-06-03 / leg. C. Bergström [CBC]. 3♂♂: 1♀, SE, UP, Nåsten / Skogsängen / O1597099 N6636152 2001-06-07 / leg. C. Bergström [CBC] (2 dissected); 2♂♂: SE, UP, Nåsten / Skogsängen / O1597099 N6636152 2005-05-13 / leg. C. Bergström [CBC]; 3♂♂: 4, 6, 7 / 2007 (red frame) // SE, UP, Uppsala, / Flogsta, Ekebydalen / O1600393 N6637642 / 2007-04-30/ leg. Christer Bergström [NHRS]; 4♂♂: 2, 5, 8, 9 / 2007 (red frame) // SE, UP, Uppsala, / Flogsta, Ekebydalen / O1600393 N6637642 / 2007-04-30/ leg. Christer Bergström [CBC]; 2♂♂: SE, VRM, Kristinehamn / O1401300 N6583100 1980-06-13 / leg. C. Bergström [CBC]; 2♀♀: SE, ÖL, S. Möckleby / ”Kalkstensbrottet” / O1537800 N6245300 2000-06-03 / leg. C. Bergström [CBC]; 1♀: SE, ÖL, Segerstad / Seby strand / O1545800 N6245500 2002-05-18 / leg. C. Bergström [CBC]; 1♂: SE, ÖL, Smedby sn. / Eckelsudde / O1537100 N6254400 2002-05-18 / leg. C. Bergström [CBC]; 4♂♂, 3♀♀: SE, ÖL, Kastlösa sn. / Bredäng / O1535100 N6260300 2002-05-19-20 / leg. C. Bergström [CBC]. **Finland**: 6♂♂, 6♀♀, including GenBank accession numbers: KX84393[MZH], KX844485, KX844383 [TMNH, rest of the specimens JPC]. **Germany**: 4♂♂, 6♀♀, Hessen and Nordrhein-Westfalen, including GenBank accession numbers: KX844199, KX843822 [both TMNH, rest of the specimens JPC].

### Tachina viridis

Taxon classificationAnimaliaDipteraTachinidae

Fallén, 1810: 276.

DCB8AEE3-1584-507D-AA86-29891C1E8E50

#### Notes.

The identity of *Tachinaviridis* Fallén, 1810: 276. *Gymnochetaviridis* (Fallén, 1810) is the oldest described *Gymnocheta* and represents the type species of the genus. The Fallén collection, drawer 13 (4), contains a single type specimen (formally the holotype) (Fig. 1B–E) mounted on an old thick pin and is in good condition except for the missing antennae and broken vibrissae. There are also some missing frontal setae and median discal setae on tergites 3 and 4. The protruding lower facial margin (Fig. 1C), metallic green femora (Fig. 1C), indistinct costal spines (Fig. 1D) and the shape of sternite 5 (Fig. 1E) make it obvious that the specimen represents the species until now known as *Gymnochetamagna* Zimin, 1958. When Fallén (1820: 25) wrote “Linea vertices subferruginea, utrinque viridi nitens. Femora virida; tibiae tarsique nigra.” [Frontal stripe brownish, both sides lustrous green. Femora green; tibiae and tarsi black.], he surely must have been looking at this specimen, as the femora of the represented species are notably green compared to the other European *Gymnocheta*. It is also obvious that the name *viridis* used by Fallén, Zetterstedt, Wahlberg, and many subsequent authors until the work of Zimin has concealed at least two different species as shown by the examination of old Swedish specimens (see below).

As a further confirmation of the type specimen identity, the original description in Swedish also refers to a single male: “♂ Denna lysande art, funnen på kummin midsommartiden i Maltesholms trädgård i Skåne, …”. [This brilliant species, found on caraway [*Carumcarvi*] in midsummer time in Maltesholms garden in Skåne, ….] (Fallén 1810: 276). In later work Fallén (1820: 25) notes records by Zetterstedt while referring to his own previous work: “Mas et Fem. In paludosis Abusa, mense Majo, utrumque sexum legit Zetterstedt. In floribus Cari Carvi in horto praedii Maltesholm mense Junio, unicum marem Muscae caesaris magnitudine vidimus ipsi.” [Males and Females. Zetterstedt collected both sexes in May [from] mires/bogs in Abusa [a place in Skåne]. In June on flowers of caraway in Maltesholm garden, a unique male observed, size of *Musca* [= *Lucilia*] *caesar*]. Zetterstedt (1844: 1190) himself writes later about *Tachinaviridis*: “Hab. in foliis fruticum & herbarum, etiam in floribus umbellatarum, locis paludosis mihi praesertim obbvia, per hortos & prata Sueciæ, 30 Maj.–15 Jun., minus frequens; scilicet in Scania ad Lund, Abusa, Maltesholm &c., passim; in templo Upsaliæ antiquæ semel a D. Prof Wahlberg capta; e Dania a D Stæger missa”. [On foliage of shrubs and herbs as well as flowers of Apiaceae, on bogs/mires [and] I especially [have observed] in gardens and meadows in Sweden. May 30–June 15, less abundant; namely from Skåne to Lund, Abusa, Maltesholm etc., infrequent; in the old Uppsala collection of D. Prof. Wahlberg; many from Denmark by D. Staeger].

Two of these aforementioned non-type specimens are in the NHRS main collection:

1♀: Labels: Hlm / P.Wg. This is apparently the specimen mentioned by Zetterstedt (1844: 1190) “in templo Upsaliae antiquae semel a D. Prof. Wahlberg capta”. This specimen, like that of the holotype, represents *Gymnochetamagna* Zimin; 1♂: Labels: Sc. // Z (on a small white tag). In contrast to the previous, this specimen represents the current concept of *Gymnochetaviridis*.

Three additional non-type specimens are present in the Diptera Scandinaviae Collection, drawer 22 in ZMLU: 1♂: Labels: lemon yellow tag // *T. viridis* / ♂. Abusa (handwritten by Zetterstedt). Identity: *Gymnochetaviridis*. This specimen is apparently one of those mentioned by Fallén (1820: 25) and by Zetterstedt (1844: 1190); 1♂: Labels: 9. // Staeger. Identity: *Gymnochetaviridis*. Zetterstedt 1844: 1190 mentioned this specimen; 1♀: Label: purple red tag // *T. viridis* / ♀. Lund. (handwritten by Zetterstedt). Identity: *Gymnochetamagna* (= *viridissensu* Fallén). This specimen is apparently the one from Lund mentioned by Zetterstedt (1844: 1190).

For the sake of nomenclatural stability, we propose maintaining the current usage of the names *G.viridis* and *G.magna*. Neotype specimen for *Gymnochetaviridis* will be assigned later if ICZN accepts the petition for the replacement of the holotype. The following redescription represents *G.viridis* in its prevailing concept.

#### Diagnosis.

A common, vivid metallic green *Gymnocheta* with strong costal spines and narrow frons in male.

#### Redescription.

Body length: 7.2–10.8 mm (n = 19).

**Male** (Figs 1A, 2C, 4C, Eiii, 5A:iii, B:iii, C:iii).

***Colouration***: Head covered with a greyish white microtomentum. The metallic ground colour subshiny at least partly on the genal dilation but the intensity of the shine depends on the direction of the incident light. Facial plate brown, in lower part sometimes with an indistinct metallic green tint. Occiput, postgena, genal dilation, ocellar tubercle metallic green in ground colour, frontal plate narrowly metallic green along the frontal setae. Palpus, clypeus (sometimes with a metallic green tint) and prementum dark brown to black, labella brown beige. Thorax and abdomen bright metallic green in ground colour, in varying degrees depending on the direction of the incident light with an indistinct light grey microtomentum. Scutum when viewed from the side and slightly from behind with four longitudinal stripes changing from grey to gilt or purple depending on the direction of the incident light. Proepisternum normally black, and densely covered with microtomentum, in larger specimens partly with a metallic green tint. Fore coxa in anterior region and femur posteriorly often with weak metallic green shine. Wing membrane around crossvein r-m not infuscated. Tegula and basicosta dark brown or black.

***Head* (Fig. 2C)**: Frons intermediate in width between *G.magna* and *G.lucida* at its narrowest point 0.35–0.45 [0.40] (n = 20) × as wide as an eye in dorsal view. Frontal vitta gradually tapering toward ocellar tubercle. Head in lateral view somewhat protruding, width at level of antennal insertion 0.60–0.77 [0.71] × (n = 14) the horizontal eye diameter. Fronto-orbital plate with a row of 10–14 [11] medioclinate moderately strong frontal setae, and some additional setulae; three or four [three to the left four to the right] of them extending to the middle of the pedicel with the row curving laterally, uppermost tiny setula sometimes reclinate; fronto-orbital plate outside the frontal row of setae with sparsely spread short and tiny setulae. Height of face 0.76–0.88 [0.85] × the length of frons (n = 13). Gena in profile at narrowest point, 030–0.42 [0.40] (n = 14) × as high as vertical eye diameter. Lower facial margin normally slightly protruded; in strict lateral view normally not visible in front of vibrissa. Facial ridge on lower 0.2–0.25 of its length with 2–3 [3] strong and 0–2 [2] additional thinner supravibrissal setae, length of which at least subequal to narrowest width of parafacial, and 2–5 [5] thin and short setulae. Below the vibrissa 4–7 [5] strong subvibrissal setae continuous to the row of genal setae, the posteriormost subvibrissal seta close to the foremost lower genal margin. Inner vertical setae crossed, thin and normally just slightly longer than the ocellar setae, outer vertical setae poorly developed, subequal with or even shorter than the adjacent postocular setae. Postocular setae long and thin, apically pronouncedly bending forward over the eyes. Occiput with a pair of postocellar setae subequal with the ocellar setae. Occiput and postgena with white to greyish white hairs. Antennae: First flagellomere subrectangular, short and evenly curved posteriorly at apex, maximal width in lateral view 0.76–0.91 [0.87] (n = 15) × the actual width of parafacial, at narrowest point; 1.47–1.73 [1.56] (n = 14) × as long as pedicel. Arista widened in proximal 2/5, rarely ½, and gradually tapering towards apex.

***Thorax***: Prosternum bare. Scutum with 3(2)+3 [3+3] acrostichal, three or four dorsocentral and 1+3 intra-alar setae. Scutellum with 4(5) pairs of strong almost horizontal setae along margin, apical and subapical pairs slightly diverging; 2–4 [2] suberect discal setae, forming a row in front of the marginal setae, the strongest subequal to the lateral setae, rarely with an additional pair of strong median discal setae; tiny dorsal setulae numerous, the longest measuring 1/2 the length of the strongest dorsal seta.

***Legs***: Claws and pulvilli on fore legs subequal in length with fifth tarsal segment, the latter 1.8–2.0 (n = 6) × as long as tarsal segment 4. Fore tibia with a row of 5–8 [five and six] anterodorsal setae, and an irregular row of 4–8 [6] posterior setae. Preapical anterodorsal seta subequal with the preapical dorsal and preapical posterior setae; preapical posterodorsal seta short, its length rarely exceeding 1/3 of the dorsal seta. Mid tibia with 4–6 [4] anterodorsal setae, the strongest in the middle of row, 5–8 [7] posterior setae, one ventral seta often accompanied by an additional tiny setula. Hind tibia with a continuous row of 9–12 unequally strong anterodorsal setae / setulae, of which 4–6 represent strong setae, four or five posterodorsal setae and three (rarely four) anteroventral setae.

***Wing***: Usually two costal spines, the lower (ventral) spine strongest and ca. 2–3 × as long as the surrounding costal setulae, normally somewhat shorter than crossvein r-m. CS_4_ + CS_5_ 2.1–2.4 (n = 12) × as long CS_6_. Vein R_4+5_ at base with 3–6 [three and four] ventral and 3–8 [4] dorsal setulae.

***Abdomen* (Fig. 4B)**: Tergite 2 with 3(2) lateral setae on each side. Tergite 3 with (2)3–4 [2 pairs and one unpaired] unequally strong and often irregularly set pairs of median discal setae; a pair of median marginal setae and 3(2) [3] lateral setae on each side. Tergites 4 with 2–3(4) [2] pairs of unequally strong and likewise irregularly set median discal setae; in dorsal view with a full row of 10–14 [10] marginal setae; tergite 5 with two or three irregular rows of strong and medium strong discal setae and a dense row of weak marginal setae.

***Terminalia*** (five dissections) (Figs 4B, Eiii, 5A:iii, B:iii, C:iii): Sternite 5 (Fig. 4B, Eiii) in ventral view with long and wide lobes; length of medial cleft ca. twice its maximal width. Lobes with widespread setulae, slightly curved inwards at apex. Dorsomedial process black and prominent, expanding backwards partly separated from the lobes. Basal plate bare, ca. 0.4 × as long as sternite 5 and 2.6–2.7 × as wide as long. Syncercus in lateral view (Fig. 5A:iii) smoothly curved at apex, in dorsal view (Fig. 5B:iii) triangular, with long basal lobes and rounded apex; 1.6–1.7 × as long as maximal width (measured from base of membranous median suture to apex), marginally incurved at middle and close to apex; numerous dorsolateral setulae at middle gives a dense and fur-like appearance similar to *G.lucida* but the setulae are slightly longer. Surstylus slender, straight in caudal view; in lateral view tapering at base and distinctly arch-like bent in proximal 1/2, apex clavate and bent towards syncercus. Pregonite (Fig. 5C:iii) in caudal view rodlike; in lateral view lobe-like, with a characteristic wide and curved hook-like projection.

Aedeagus: Distiphallus in lateral view of almost uniform width.

**Female** (Figs 3C, 6A:iii, B:iii): Differs from male as follows:

***Colouration***: Fronto-orbital plate vivid metallic green when viewed from behind and slightly from above; the metallic ground colour continues down on the upper part of parafacial reaching the level of the lowermost frontal setae; the upper part of parafacial and anterior 1/3 of fronto-orbital plate with a thin greyish white microtomentum, visible along eye margin and as a slim line at edge of frontal vitta. Thorax and abdomen vivid metallic green at most with a thin whitish microtomentum.

***Head*** (Fig. 3C): Frons wider, at its narrowest point 0.76–0.93 (n = 12) × as wide as an eye in dorsal view. Frontal vitta gradually tapering towards ocellar tubercle, at this level often narrower than width of fronto-orbital plate. Fronto-orbital plate normally with fewer, 8–11 moderately strong medioclinate frontal setae. Outer vertical setae subequal with the ocellar setae, shorter than the posteriormost proclinate orbital setae, at least 2 × the length of the adjacent postocular setae. Postocellar setae short and tiny and subequal with the upper postocular setae. First flagellomere 1.38–1.62 (n = 8) × as long as pedicel; its maximum width 0.82–0.93 of the width of the parafacial at narrowest point.

***Legs***: Claws and pulvilli on fore legs shorter ca. 0.7–0.8 × as long as tarsal segment 5, the latter 1.5–1.7 × as long as tarsal segment 4.

***Abdomen***: Tergites 3 and 4 normally with two pairs of median discal setae, tergite 3 rarely missing one seta or with one additional seta, tergite 4 occasionally devoid of one seta. Ground vestiture dorsolateral on tergites 3 and 4 distinctly prone contrasting to the erect setulae between the median discal setae. Tergite 5 trapezoid, along anterior margin ca. 2 × as wide as long. Sternite 5 ca. as long as its maximal width, with 6–10 strong setae in posterior 1/3, four of them along the posterior margin and one or two pairs of median setae, in posterior 1/2 accompanied by numerous (60–70) irregularly spread setulae of varying size.

***Terminalia*** (four dissections) (Fig. 6A:iii, B:iii): Tergite 6 in form of widely separated hemitergites; without tiny setulae in the adjacent posterior membranous area. Sternite 6 (Fig. 6A:iii) shorter 0.6–0.7 × as long as wide, slightly wider (1.1–1.2 ×) than sternite 5 and 1.8–1.9 × as wide as long, with 6–8 relatively strong setae along the posterior margin, accompanied by numerous (>20) tiny setulae. Tergite 7 strongly reduced, without tiny setulae in the adjacent membranous area. Sternite 7 slightly narrower than sternite 6, with numerous tiny setulae most of them restricted to the posterior margin. Sternite 8 reduced to a small faintly sclerotised plate with 6–12 sensory pores (sensilla trichodea). Hypoproct (Fig. 6B:iii) in ventral view distinctly rounded at apex (studied in ventral and somewhat caudal position somewhat blunt at apex): with a prominent depression, densely covered with short and tiny setulae, only a few in lateral position longer than the hypoproct itself; in lateral view not curved towards the cerci, apex of hypoproct reaching tip of cerci, lingulae well developed. Epiproct strongly reduced but in caudal view normally indicated by the presence of 2–4 setulae inserted between the cerci and above the hypoproct. Cerci reminiscent of a short-shafted paddle, weakly sclerotised with numerous setulae of varying length, in lateral view with some setulae that are subequal to the length of sternite 7 (twice the length of cerci itself).

#### DNA.

The European specimens of *G.viridis* share the Co1 DNA barcode sequence (BOLD:ACF3891) with Finnish *G.zhelochovtsevi* (Fig. 7).

#### Distribution.

A widely distributed and common species in the Palaearctic Region, including all of Europe, Japan (Hokkaido, Honshu), Middle East, all of Russia and Transcaucasia (Richter 2004; O’Hara et al. 2009). It is possible that at least some northern records of the species, at least from the Taiga region in the Nordic countries, represent *G.lucida*.

#### Biology.

*Gymnochetaviridis* is a common spring–early summer species, often numerous at the right locations. Typical habitats include forest margins and meadows, but the species is frequently seen also in gardens. The males start their flight in early April in Central Europe and around end of April to early June in the north. The last female records in Finland are from mid-July. Males are frequently observed basking on tree trunks and watching for passing females. Both sexes can be collected from flowers, especially on cow parsnip (*Anthriscussylvestris*), but also from other Daucaceae. The species is a known parasitoid of Erebidae (Lepidoptera) living in grass tufts, such as *Mesapameasecalis* Linnaeus and *Photedesminima* Haworth (Tschorsnig 2017). The females can be frequently seen in meandering flight in the low vegetation and investigating tufts of grass or sedges in search for a host.

### Gymnocheta zhelochovtsevi

Taxon classificationAnimaliaDipteraTachinidae

Zimin

749C91A3-CBC6-5F1C-B5BC-8A104E82E408

Gymnocheta zhelochovtsevi Zimin, 1958: 62. Holotype ♂ [ZIN] (see below).

#### Material.

**Russia**: ♂: Южные Курилы, Итуруп, Рыбаки, 5 км SW Курильска, В. Рихтер, 23. VI.1968 [Southern Kurils, Iturup, Ribaki, 5 km SW Kurilsk, V. Richter, 23. VI.1968], det. V. Richter. Dissected by V. Richter [ZIN]. Examined from high quality photographs, including the terminalia. **Japan**: ♂: JAPAN / Mt. Muine / Sapporo / HOKKAIDO / 20 Jun.1974 / K. Nishida // *Gymnochaeta* / *zhelochovtsevi* ? / Zimin (handwritten) / det. H. Shima 1982 [BLKU]; ♂: JAPAN / Hokkaido / Churui Vil. Haruyama / 1 Jul.1993 / A. Kuromoto // *Gymnocheta* ♂ / *zhelochovtsevi* Zimin / det. C. Bergström 2020 [BLKU]. Dissected. **Finland**: ♂: Regio aboensis, Salo, Halikko, Perkko, 67158-60:32853-8, 12.vi.2020, A. Haarto leg. [AHC], BOLD Sample-ID JP2020-G1; ♂: Karelia australis, Vehkalahti, 18.vi.1966, L. Tiensuu leg. Dissected. [MZH]; ♀: Regio aboensis, Korppoo, 66840:31958, malaise, 19.vi.–22.vii.2003. A. Haarto leg. Dissected. BOLD Sample-ID JP2020-G2; 2♀♀: Regio aboensis, Salo, Halikko, Perkko, 671604:328551, 12.-28.vi.2020, malaise, Haarto leg. [AHC]; 1♀: Regio aboensis, Salo, Halikko, Perkko, 67158-60:32853-8, 28.vi.2020, A. Haarto leg. [AHC]. **Sweden**: 1♀: S. Vb: Umeå: Tavelån: / Östra Tjälamark; malaise / RT 90 70952, 17177 / 10.v.-16.vi.2016 / S. Hellqvist 14947 // *Gymnocheta* ♀ / *zhelochovtsevi* Zimin / det. C. Bergström 2020 [CBC].

#### Diagnosis.

*Gymnochetazhelochovtsevi* is characterised by a dark, almost black metallic green, wide frons in both sexes, and a hooked syncercus in the male.

**Redescription.** Body length: 9.2–10.4 mm (n = 7).

**Male** (Figs 2D, 4C, Eiv, 5A:iv, B:iv, C:iv).

***Colouration***: Head covered with dense greyish or yellowish white microtomentum. The metallic ground colour is only weakly shining through, similar as is seen with *G.lucida*. Facial plate black without a metallic green spot. Occiput, postgena, genal dilation, ocellar triangle and frontal plate almost black in ground colour, although a hint of dark metallic bronze-green shine can be seen with changing light incidence. Palpus clypeus and prementum black, labella dark brown. Thorax and abdomen dark metallic green in ground colour, not as lucid as in *G.magna* or *G.viridis* and covered with indistinct greyish white microtomentum. Scutum, when viewed from the side and slightly from behind, with four pronounced longitudinal stripes of microtomentum, changing from grey to purple depending on the direction of the incident light. Proepisternum black and with thin grey microtomentum. Legs extensively black, but fore coxa in anterodorsal region and sometimes also femora with remnants of metallic shine, covered with light grey microtomentum. Wing membrane around crossvein r-m not infuscated.

***Head* (Fig. 2A)**: Frons at its narrowest point, 0.53–0.68 (n = 5) × as wide as an eye in dorsal view. Frontal vitta almost parallel-sided in anterior part hardly tapering until closely before ocellar tubercle. Head in profile protruding at level of antennal insertion, width of parafacial at this level ca. 0.65–0.75 × (n = 2) the horizontal eye diameter. Fronto-orbital plate with a row of 10–12 medioclinate moderately strong frontal setae and some additional setulae, uppermost setula tiny and sometimes slightly reclinate, four or five setae extend to the middle of the pedicel with the row curving laterally; fronto-orbital plate outside the frontal row of setae with 4–10 sparsely spread, short and tiny setulae. Height of face 0.8–0.9 × the length of frons (n = 2). Gena in profile at narrowest point, 0.4–0.5 (n = 2) × as high as vertical eye diameter. Facial plate and lower facial margin not visible in profile. Facial ridge on less than lower 1/5 with one or two strong, two additional thinner supravibrissal setae, and four or five setulae. Ridge below vibrissa with 3–5 strong subvibrissal setae continuous with the genal setae, longest ca. 0.6 × the length of vibrissa. Inner vertical setae strong and crossed, longer than the ocellar setae; outer vertical setae subequal with inner vertical setae, distinctly stronger than the postocular setae. Postocular setae short and barely bending forward over the eyes. Occiput with a pair of postocellar setae, weaker that the outer vertical setae, thinner but longer than the postocular setae. Palpus slightly clavate at tip, subequal to the length of the antenna and densely covered with short black setulae, two strong preapical setulae and 4–5 irregular ventral setulae. Antenna: Pedicel subtriangular and with one elongate seta, 1.2–1.5 × as long as wide at apex (n = 4). First flagellomere 1.5–1.7 × (n = 5) as long as the pedicel, in profile subrectangular, rounded at apex; maximal width in profile as wide (0.9–1.1) (n = 2) as parafacial at narrowest point. Eyes sparsely covered with, compared with the other species, relatively short whitish hairs with a yellowish tint.

***Thorax***: Prosternum bare. 2(3)+3 acrostichal, 3+4 dorsocentral and 1+3 intra-alar setae. Ground vestiture on scutum (consisting of thin setulae) sparse and erect, longest setulae 0.5–0.6 × as long as the shortest setae, anteriorly shorter, at most 0.3 × the length of the setae. Scutellum with four pairs of strong marginal setae, mixed with some shorter and weaker marginal setae/setulae, almost horizontal with the plane of scutellum, apical setae missing; subapical setae close to the apex, parallel or slightly diverging, two lateral pairs and one basal pair; four suberect preapical discal setae, forming a row in front of the marginal setae, the strongest pair in the middle sometimes subequal to the lateral setae, mixed with numerous tiny setulae the longest at least 1/2 as long as the strong preapical setae.

***Legs***: Claws and pulvilli on fore legs ca. 1.0 (n = 3) × as long as tarsal segment 5, the latter 2.0 (n = 3) × as long as tarsal segment 4. Fore tibia with a row of 5–7 anterodorsal setae, 3–5 setae in an irregular posterodorsal row and two posterior setae; preapical anterodorsal seta well developed, subequal with the preapical dorsal and preapical posterior setae. Mid tibia with a row of five anterodorsal setae the strongest in the middle, four or five posterodorsal setae, two or three posterior setae, one strong ventral seta accompanied by a shorter seta above. Preapical anterodorsal seta of the hind tibia subequal to the preapical dorsal seta, preapical anteroventral seta 1/2 the length of anterodorsal seta; a continuous irregular row of 8–10 anterodorsal setae, three or four posterodorsal setae and three or four anteroventral setae.

***Wing***: One or two costal spines, the strongest lower spine 2–3 × as long as the surrounding costal setulae. Fourth and fifth costal section ca. 2 × as long as sixth costal sector. Vein R_4+5_ at node with four ventral and three or four dorsal setulae. Cell r_4+5_ often somewhat narrow at wing edge, 0.8 × the length of crossvein r-m.

***Abdomen* (Fig. 4C)**: Domed, ground-vestiture prone or semierect on tergite 5, also ventrally. Tergite 2 with two lateral setae on each side. Tergite 3 with two pairs of median discal setae; with a pair of median marginal setae and two or three lateral setae on each side. Tergite 4 similarly with two or three pairs of unequally and irregularly set strong median discal setae, in dorsal view with a full row of 10–12 marginal setae. Tergite 5 with two or three irregular rows of unequally strong discal setae and a row of strong marginal setae.

***Terminalia*** (three dissections) (Figs 4C, Eiv, 5A:iv, B:iv, C:iv):

Sternite 5 (Fig. 4C, Eiv) in ventral view with long and wide lobes; length of medial cleft 1.3–1.4 × its maximal width. Lobes with numerous setulae, apex in ventral view rounded in a ventral and slightly lateral view with a subtriangular appearance. Dorsomedial process long and narrow, indistinct, and visible only as a callosity, along its length connected with the lobes. Basal plate bare, ca. 0.4 × as long as sternite 5 and 2.6–2.7 × as wide as long. Syncercus in profile (Fig. 5A:iv) dorsally strongly bowed in the middle and with a characteristic tooth-like hook at the apex, reminiscing of the situation in *Gymnochetaporphyrophora*Zimin (1958); in caudal view (Fig. 5B:iv) subtriangular with long basal lobes and a widened apex; dorsolateral with numerous long setulae that generate a dense and fur-like hairiness. Surstylus (Fig. 5A:iv) bacilliform, straight in caudal view, in profile gradually tapering at base, then slightly arch-like bent at middle; evenly thick apart from an indicated widening at apex, bent towards syncercus. Pregonite rodlike in caudal view; lobe-like in profile (Fig. 5C:iv) with a wide hook-like projection apically, shortly tapering at apex. Aedeagus: Basiphallus with a basal projection and a distinct digitiform epiphallus, resembling the preceding species. Distiphallus (Fig. 5C:iv) in profile compact and evenly wide.

**Female** (Figs 3D, 6A:iv, B:iv): Differs from male as follows:

***Colouration***: Fronto-orbital plate almost entirely metallic dark green when viewed from behind and slightly from above; the metallic ground colour at least partly interrupted on the upper part of parafacial. However, most of the metallic ground colour on upper part of parafacial and anterior 1/3 of fronto-orbital plate is covered by greyish white microtomentum, which is dense especially along eye margin and frontal setae.

***Head* (Fig. 3D)**: Frons wider, at its narrowest point 0.8–1.0 (n = 4) × as wide as an eye in dorsal view. Frontal vitta wide and with parallel edges in anterior 1/2 and then slightly tapering towards ocellar tubercle, its width at ocellar tubercle exceeding the width of fronto-orbital plate at this point. Fronto-orbital plate normally with fewer, 6–9 moderately strong medioclinate frontal setae. Outer vertical setae strong and 0.6–0.7 × the length of inner vertical setae, stronger than ocellar setae. Postocellar setae subequal with the upper postocular setae. First flagellomere 1.3–1.5 (n = 4) × as long as pedicel, normally as wide as, or slightly wider than the parafacial at narrowest point.

***Thorax***: Whitish grey microtomentum normally thinner than in males, most intense on the pleura and episternum.

***Legs***: Claws and pulvilli on fore legs shorter ca. 0.7–0.8 (n = 5) × as long as tarsal segment 5, the latter 1.5–1.6 (n = 5) × as long as tarsal segment 4.

***Abdomen***: Tergites 3 and 4 with one or two pairs of median discal setae. Tergite 5 trapezoid, along anterior margin ca. 2 × as wide as long, posterior edge concave (blunt or pointed in *G.lucida*). Sternite 5 slightly wider than long, with 4 strong setae along posterior margin (Fig. 6A:iv).

***Terminalia*** (one dissection) (Fig. 6A:i, B:i): Tergite 6 divided into narrow and somewhat elongated hemitergites, widely separated, each closely accompanied by a pair of setulae at posterior margin. Sternite 6 shorter, 0.5 × as long and 1.2 × as wide as sternite 5 (n = 1), with ca. 10 tiny setulae along the posterior margin. Tergite 7 strongly reduced. Sternite 7 semi-spherical, ca. as long but slightly narrower than sternite 6, with numerous setulae most of them tiny, restricted to the posterior margin. Sternite 8 strongly reduced, partly hidden below sternite 7. Hypoproct (Fig. 6B:iv) in ventral view almost triangular, pointed, apex only slightly rounded, densely covered with setulae that are longer laterally; in profile slightly bent towards the cerci, apex of hypoproct not reaching the tip of cerci. Cerci elongated, with numerous setulae of varying length, the strongest setulae in profile subequal to the length of sternite 7.

#### DNA.

Despite the morphological differences, *G.zhelochovtsevi* shares Co1 DNA barcode sequence with *G.viridis* (Fig. 7). DNA barcode sharing is not uncommon in Tachinidae (Pohjoismäki et al. 2016).

#### Distribution.

Like *G.lucida*, *G.zhelochovtsevi* had been thought to be an Eastern Palaearctic species (Richter 2004). So far, the only records from Europe are the ones listed here from the southern and southwestern coast of Finland and from Sweden.

#### Biology.

The specimens from Halikko were collected from a flood meadow adjacent to a meandering stream. The meadow is characterised mainly by tall grass and sedges, surrounded by shrub of willows (*Salix* spp.) and bird cherry (*Prunuspadus* L.). One male and one female were hand netted from the flowers of *Anthriscussylvestris* (L.) and two females were collected with a Malaise trap. The female specimen from Korppoo was caught in a Malaise trap in a seashore meadow, surrounded by shrub of meadowsweet (*Filipendulaulmaria* (L.)) and alders (*Alnus* spp.). The Swedish specimen was collected in a Malaise trap, placed close to the calmly flowing, slightly meandering, ca. 5 m wide Tavelån River. The trap was placed in overgrowth known as “raningsmark”; i.e., grassland long ago used as a hay meadow or grazing but now characterised by bush wood with meadow glades between the shrubbery.

#### Notes.

Lauri Tiensuu mentioned on page 138 of his unpublished notes that a specimen identified by him as a female of *G.viridis*, but actually a male of *G.zhelochovtsevi*, was collected around (on?) aspen (*Populustremula* L.) trunks near the village of Salmenkylä in the former municipality of Vehkalahti. The original description by Zimin was based on one male specimen, whose fifth sternite was poorly illustrated and the epandrium not illustrated at all in the original publication. The tip of the syncercus of the species has a characteristic hook (Figs 4C, 5A:iv), somewhat resembling that of *Gymnochetaporphyrophora*, which caused us some confusion. However, the median lobes at the posterior edge of the sternite of *G.porphyrophora* are well developed (Fig. 4D), unlike in any of the other species discussed here.

### Revised key to the Palaearctic *Gymnocheta* species

This identification key is based on the ones provided by Zimin (1958) and Richter (2004) with some modifications. The extent of microtomentum at the orbits and upper part of the frons as described by Zimin (1958) and Mesnil (1975) differentiating *Gymnochetamagna* and *G.viridis* is not reliable on its own, especially in females. Although on average specimens of *G.lucida* and *G.zhelochovtsevi* can be differentiated from *G.magna* and *G.viridis* by their colouration, we do not regard the colour alone an important diagnostic feature as it can be influenced by the developmental conditions as well as by the age and condition of the specimen. However, the colour difference can be useful when sorting out specimens for closer inspection. At first glance, the colouration of *G.lucida* appears quite matt, olive metallic green and that of *G.zhelochovtsevi* very dark green compared to the bright, almost golden metallic green of *G.magna* and *G.viridis*. As we do not have much knowledge of *G.goniata*, apart from the rather short description (Chao 1979), it is not included in the key. Based on the figures in Chao (1979: figs 6, 7), the species seems to be closely related to *G.flamma*, when comparing the shape of sternite 5 and the hooked syncercus (not broadened dorsally at the tip as in *G.zhelochovtsevi*). The species is bright green in contrast to the cherry-red *G.flamma*.

**Table:** 

1	Cell r_4+5_ short-stalked, closed, or rarely narrowly open at wing margin; appendage at bend of vein M equal to or slightly shorter than distance between crossvein dm-cu and bend; body slender, shining, dark olive green; length of external costal spine equal to length of crossvein r-m, width of frons > 1/2 as wide as an eye [Not seen. Female unknown]	***Gymnochetamesnili* Zimin**
–	Cell r_4+5_ open, other combinations of features	**2**
2	Abdomen cherry-red or wine red	**3**
–	Abdomen green	**4**
3	Scutum with 4 brownish black, distinct narrow longitudinal vittae separated by wide areas of thin white microtomentum, distinct in the presutural part of scutum. Frons in dorsal view 0.25–0.33 × the width of an eye in males and 0.66 × in females. Lower facial margin in lateral view distinctly protruding between the vibrissae. Male sternite 5 as in Fig. 4D, syncercus with an apical hook similar to Fig. 5A:iv	***Gymnochetaporphyrophora* Zimin**
–	Frons in male narrow, 0.20–0.25 × the width of an eye. Apex of syncercus not wider than apex of surstylus (caudal view), labella small-sized [Not seen. Female unknown]	***Gymnochetaflamma* Zimin**
4	Males	**5**
–	Females	**8**
5	Frons in dorsal view 0.5–0.7 × the width of an eye (Fig. 2D). Frontal vitta wide, at level of ocellar tubercle 3.0–3.5 × the width of the orbital plate. Fronto-orbital plate, parafacial, genal dilation and occiput coated by a dense greyish white microtomentum. Outer vertical seta almost as strong as the inner vertical seta, much stronger than the postocellar setae. Thorax and abdomen of a dark metal green colour. Terminalia as in Figs 4C, E:iv, 5A:iv, B:iv, C:iv	***Gymnochetazhelochovtsevi* Zimin**
–	Frons in dorsal view either not exceeding 0.4 × the width of an eye, and frontal vitta gradually narrowing towards ocellar tubercle (*G.magna* and *G.viridis*), or frons wider (*G.lucida*), but then frontal vitta narrowing towards middle. Frontal vitta at level of ocellar tubercle at most twice the width of the orbital plate. Occiput at least mediodorsally with a shiny metallic green colour. Outer vertical seta weaker than the inner, subequal to the postocellar setae	**6**
6	Frons in dorsal view at its narrowest point, 0.43–0.51 × width of an eye (Fig. 2A). Frontal vitta narrowing towards middle, then edges parallel reaching ocellar tubercle. Fronto-orbital plate, parafacial, and genal dilation with a rather dense greyish white microtomentum, the metallic green colour almost not shining through. Thorax and abdomen olive green. Scutum with thin microtomentum, when viewed from above and slightly from behind with distinct greyish white longitudinal stripes. Arista widened in at least its basal 1/2, sometimes almost in basal 2/3 and gradually tapering to apex. Proepisternum always black and densely covered with microtomentum Terminalia as in Figs 4A, E:i, 5A:i, B:i, C:i	***Gymnochetalucida* Zimin**
–	Frons in dorsal view rarely exceeding 0.4 × width of an eye. Frontal vitta gradually narrowing towards ocellar tubercle. Fronto-orbital plate, parafacial, and genal dilation with a thin greyish white or white microtomentum but the metallic green colour subshiny at least at the fronto-orbital plate. Thorax and abdomen more bright green, sometimes with a golden- or blue green lustre. Scutum with thin microtomentum. Arista normally widened in less than proximal 1/2. Proepisternum usually partly metallic green	**7**
7	Lower facial margin slightly protruding (Fig. 2C), normally without areas of a green shine. Costal spine distinct, at least 0.7 × the length of crossvein r-m. Arista widened in proximal 2/5, rarely 1/2 of its length. Terminalia as in Figs 4C, E:iii, 5A:iii, B:iii, C:iii	***Gymnochetaviridis* (Fallén)**
–	Lower facial margin strongly or moderately protruding (Figs 1C, 2B). Costal spine short, 0.5 × the length of crossvein r-m. Arista widened in its proximal 1/4–1/3. Terminalia as in Figs 4E:ii, 5A:ii, B:ii, C:ii	***Gymnochetamagna* Zimin**
8	Frons in dorsal view 0.8–1.0 × as wide as an eye (Fig. 3D). Frontal vitta wide, with almost parallel edges in ca. anterior 1/2, then gradually tapering towards ocellar tubercle; width at level of ocellar tubercle subequal to the orbital plate. Scutum with 4 greyish white longitudinal stripes due to the dense microtomentum covering the dark stripes, present also behind the transverse suture. Postpronotum and scutellum also with distinct greyish white microtomentum. Occiput covered with a dense greyish microtomentum. Parafacial, genal dilation and fronto-orbital plate in anterior 1/3 and along eye margin with greyish white microtomentum. Thorax and abdomen dark metallic green. Terminalia as in Figs 6A:iv, B:iv	***Gymnochetazhelochovtsevi* Zimin**
–	Frons normally not exceeding 0.9 × the width of an eye. Frontal vitta either gradually tapering towards ocellar tubercle or narrowest at ca. middle. Scutum either with 4 greyish white longitudinal stripes or when viewed from different directions with 4 dark stripes covered by thin microtomentum. Occiput in upper part behind the row of postocular setulae and fronto-orbital plate at least in their upper part metallic green, without microtomentum	**9**
9	Thorax and abdomen metallic olive green; viewed from different directions notably scutum with a bronze or golden lustre. The greyish white microtomentum in anterior part of fronto-orbital plate shifting into a bronze or golden lustre when viewed from different directions. Frontal vitta tapering towards middle, then slightly widening towards ocellar tubercle. Width of first flagellomere normally wider than parafacial at narrowest point. Arista widened in at least its basal 1/2, sometimes almost in proximal 2/3 and gradually tapering to apex. Cell r_4+5_ narrowly open, or (as an exception) closed at wing margin. Terminalia as in Figs 6A:i, B:I	***Gymnochetalucida* Zimin**
–	Thorax and abdomen bright metallic green; viewed from different directions with purple lustre. The greyish white microtomentum in anterior part of fronto-orbital plate sometimes shifting into a silvery lustre. Frontal vitta gradually tapering towards ocellar tubercle. Width of first flagellomere not exceeding width of parafacial at narrowest point. Arista normally widened in less than proximal 1/2. Cell r_4+5_ wide open at wing margin	**10**
10	Lower facial margin hardly protruding between vibrissae (Fig. 3C). Costal seta at least 0.7 × the length of crossvein r-m. Prosternum without setulae. Arista widened in proximal 2/5 (or rarely 1/2) and gradually tapering towards apex. Terminalia as in Figs 6A:iii, B:iii	***Gymnochetaviridis* (Fallén)**
–	Lower facial margin distinctly protruding between vibrissae (Fig. 3B). Costal seta short, at most 0.5 × the length of crossvein r-m. Prosternum sometimes with setulae. Arista widened in its proximal 1/4–1/3, gradually tapering towards apex. Terminalia as in Fig. 6A:ii, B:ii	**Gymnochetamagna Zimin**

## Supplementary Material

XML Treatment for Gymnocheta

XML Treatment for Gymnocheta lucida

XML Treatment for Gymnocheta magna

XML Treatment for Gymnocheta viridis

XML Treatment for Tachina viridis

XML Treatment for Gymnocheta zhelochovtsevi
